# Exploring the operating factors controlling *Kouleothrix* (type 1851), the dominant filamentous bacterial population, in a full-scale A2O plant

**DOI:** 10.1038/s41598-020-63534-2

**Published:** 2020-04-22

**Authors:** Tadashi Nittami, Risa Kasakura, Toshimasa Kobayashi, Kota Suzuki, Yusuke Koshiba, Junji Fukuda, Minoru Takeda, Tomohiro Tobino, Futoshi Kurisu, Daniel Rice, Steve Petrovski, Robert J. Seviour

**Affiliations:** 1 Division of Materials Science and Chemical Engineering, Faculty of Engineering, Yokohama National University, 79-5 Tokiwadai, Hodogaya-ku, Yokohama 240-8501 Japan; 2 Department of Chemistry, Chemical Engineering and Life Science, College of Engineering Science, Yokohama National University, 79-5 Tokiwadai, Hodogaya-ku, Yokohama 240-8501 Japan; 3 Department of Urban Engineering, Graduate School of Engineering, The University of Tokyo, 7-3-1 Hongo, Bunkyo-ku, Tokyo 113-8656 Japan; 4 Collaborative Research Institute for Innovative Microbiology, The University of Tokyo, 1-1-1 Yayoi, Bunkyo-ku, Tokyo 113-8657 Japan; 5 Research Center for Water Environment Technology, The University of Tokyo, 7-3-1 Hongo, Bunkyo-ku, Tokyo 113-8656 Japan; 6 Department of Physiology, Anatomy, and Microbiology, La Trobe University, Bundoora, VIC3086 Australia

**Keywords:** Environmental biotechnology, Water microbiology, Microbial ecology

## Abstract

This study reveals that the abundance of the filament *Kouleothrix* (Eikelboom type 1851) correlated positively with poor settleability of activated sludge biomass in a Japanese full-scale nutrient removal wastewater treatment plant sampled over a one-year period. 16S rRNA amplicon sequence data confirmed that *Kouleothrix* was the dominant filament in the plant, with a relative abundance of 3.06% positively correlated with sludge volume index (SVI) (*R* = 0.691). Moreover, *Kouleothrix* (type 1851) appeared to form interfloc bridges, typical of bulking sludge, regardless of season. Together with earlier studies that indicated the responsibility of *Kouleothrix* (type 1851) on bulking events, these data suggest that their high relative abundances alone may be responsible for sludge bulking. 16S rRNA qPCR data for this filament showed changes in its relative abundance correlated with changes in several operational parameters, including mixed liquor temperature, sludge retention time, and suspended solids concentration, and it may be that manipulating these may help control *Kouleothrix* bulking.

## Introduction

The activated sludge process is the most popular method worldwide for the biological treatment of both municipal and industrial wastewaters. In conventional systems, solid-liquid phase separation is performed to produce ideally a final clear treated liquid effluent from the secondary settling tank^1^. Activated sludge bulking in the secondary settling tanks is a serious operational problem, which can lead to an outflow of suspended solids from the tank and decreasing biomass concentration in the reactor, resulting in a poor quality effluent, with poor thickening and dewatering characteristics^1^. Bulking is caused usually by excessive proliferation of filamentous bacteria, which appear to interfere with floc sedimentation and compaction^2^.

Existing filamentous bulking control methods can be considered as specific and non-specific^3^. The latter usually consist of adding chemicals, including oxidizing agents (chlorine, ozone, hydrogen peroxide), weighting and flocculating agents (salts of iron and aluminium, lime, polymers and talc), and biocides^3^. These methods provide only temporary relief. They are useful when the cause of the filamentous bulking cannot be determined immediately, and when rapid resolution of a bulking problem is necessary^3^. On the other hand, specific targeted control methods if available are more attractive since high performing plants can operate with a limited filament proliferation^3^. Although the problem of bulking has attracted considerable interest, conclusions as to which individual ecological and physiological factors might favor excessive filament growth are still not available^3^. Quantitative PCR (qPCR) assays have identified correlations between “*Ca*. Microthrix parvicella”^4–6^, *Thiothrix eikelboomii*^7,8^, *Sphaerotilus natans*^9^ and *Haliscomenobacter hydrossis*^10^ 16 S rRNA gene copy numbers and sludge settleability. However, such quantitative studies are rare.

We have shown that relative percentage abundances of type 1851/ *Kouleothrix* had a quantitative linear relationship with biomass settleablity in several municipal wastewater treatment plants (WWTPs) in Yokohama, especially during the summer months^11^. More recently this approach has been extended to quantify *Kouleothrix* in 11 municipal WWTPs across Japan, and the data generated confirmed that increases in their abundances corresponded to sludge bulking episodes^12^. Thus, *Kouleothrix* abundance (>10^5^ 16S rRNA gene copies ng-DNA^−1^) almost always coincided with poor activated sludge settleability (i.e., sludge volume index (SVI) > 200 mL g-sludge^−1^). In some plants, *Kouleothrix* spp. were not always as highly abundant, but our view was that phase separations would improve if their numbers could be suppressed because of their propensity to participate in interfloc bridging^12,13^. However, both these studies examined samples collected mainly during summer. Therefore, in this present study, additional samples were taken regularly over a 12-month period to clarify further the relationship between *Kouleothrix* relative abundance and SVI.

Nittami *et al*. (2019)^12^ also compared the average 16S rRNA gene copy numbers of *Kouleothrix* in samples from plants of different process configurations. These were conventional activated sludge (C), phosphorus removal (P) nitrogen removal (N) and both P and N removal (N&P) processes. They showed those for C, P, and N&P removal plants all had high average gene copy numbers for *Kouleothrix*, but often with high standard deviations. In particular, N&P processes had the highest average copy number (1.01 × 10^5^ 16S rRNA gene copies ng-DNA^−1^) but with the highest standard deviations (7.67 × 10^4^). However, such trends seemed to suggest that relative abundances of *Kouleothrix* spp. in N&P removal may reflect plant operating conditions.

Here we focus on the relationship between *Kouleothrix* filamentous bacteria and SVI in a Japanese municipal WWTP N&P removal (i.e., anaerobic-anoxic-aerobic (A2O) process) to see how their contribution to sludge settleability varied over a 12 month period. To assess their contribution to sludge settleability, the identity of other putative bulking bacteria present in this microbial community was sought and their contributions if any, to bulking examined. In addition, correlations between their relative abundances and environmental/operational conditions were analyzed statistically to explore which factors might affect their abundances.

## Materials and Methods

### Sample fixation, DNA extraction, and qPCR

Activated sludge-mixed liquor samples were collected from an independent A2O process train with about 3,000 m^3^ of effective reaction-tank capacity, located at a municipal WWTP in Japan (percentage of municipal wastewater >90%) every 1–2 weeks for a year from Sept 2016 to Sept 2017. Grab samples were taken at the end of the aeration tank. An aliquot of mixed liquor of each sample was fixed in either 4% (w/v) paraformaldehyde (PFA) or 96% (v/v) ethanol and then stored at −20 °C before FISH analysis according to the methods described by Daims *et al*. (2005)^14^. A 1 mL aliquot of mixed liquor from each sample was washed with Tris-EDTA (T_10_E_1_, pH 8.0) buffer on the sampling day and stored at −20 °C prior to DNA extraction. DNA was extracted using the ISOIL for Beads Beating kit (Soil DNA Extraction Kit, Nippon Gene, Tokyo, Japan) as detailed previously^11,12^. The 16S rRNA genes of *Kouleothrix* were amplified and quantified by real-time qPCR according to the methods used previously^11^. The PCR mixture (20 μL) contained 10 μL of THUNDERBIRD^®^ SYBR qPCR Mix (TOYOBO, Osaka, Japan), 2 μL (0.5 pmol) of each primer (CHL1851_658f and CHL1851_815r), 1 μL (30 ng) of template DNA, and 5 μL of sterilized milliQ water. Each amplification was performed in triplicate using a Thermal Cycler Dice Real Time System TP800 (Takara Bio, Shiga, Japan) under the optimized conditions of Nittami *et al*. (2017)^11^. Standard curves were constructed with plasmid DNA possessing the 16S rRNA gene of *Kouleothri*x B09 clone sequence as described previously^11^. The 16S rRNA gene copy numbers ranged from 2.28 × 10^2^ copies ng-DNA^−1^ to 2.28 × 10^6^ copies ng-DNA^−1^ where the average amplification efficiencies showed 97.7% (S.D. = 0.6%) with an average linear slope of −3.38 (S.D. = 0.01). In the dissociation curve analyses, only a single peak was detected.

### Amplicon sequencing

The extracted DNA samples were sent to the Bioengineering Lab. Co., Ltd. (Atsugi, Japan) and amplicon sequencing and data processing work were conducted there. The amplicon libraries were prepared using a two-step tailed PCR method. Briefly, the 1^st^ PCR amplified V3 − V4 region of 16S rRNA gene using primers 341f (ACACTCTTTCCCTACACGACGCTCTTCCGATCT – NNNNN – CCTACGGGNGGCWGCAG) and 805r (GTGACTGGAGTTCAGACGTGTGCTCTTCCGATCT – NNNNN – GACTACHVGGGTATCTAATCC) and the 2^nd^ PCR was performed using the 1^st^ PCR products and index primers. Amplicon sequencing of the 2^nd^ PCR products (i.e., amplicon libraries) was carried out, with paired-ending (2 × 300 bp) on an Illumina MiSeq platform (San Diego, CA, USA). Sequencing data were analyzed using the QIIME2 software package version 2019.01^15^. Briefly, primers were trimmed and data were filtered and truncated, to allow 20 bp overlap between forward and reverse reads, using Dada2. Possible chimeric sequences were identified and removed before amplicon sequence variants were assigned using MiDAS database (Version 2.1.3)^16^. Raw sequence data were deposited in the DDBJ Sequence Read Archive (DRA) under the number DRA008490.

### FISH analyses

FISH analyses were performed to identify *Kouleothrix* present in the samples using existing oligonucleotide probes: CHL1851^13^ labeled with Cy5, targeting *Kouleothrix* (type 1851). Other probes applied were the EUBmix probes targeting most bacteria^17,18^, the T0803-654 probe targeting “*Ca*. Defluviifilum” (type 0803)^19^, and the HGC69a (together with its unlabelled competitor probe) for the Gram positive bacteria, which include *Tetrasphaera jenkinsii* (*Nostocoida limicola* II)^20^. Probes were tagged with Fluos, Cy3, and Cy3 fluorochromes, respectively. Probes labeled with Fluos and Cyanines (i.e., Cy3 and Cy5) fluorochromes were purchased from Rikaken (Nagoya, Japan) and Proligo (Sydney, NSW), respectively. The PFA and ethanol fixed samples were hybridized with EUBmix, CHL1851, and T0803-654, and EUBmix, CHL1851, and HGC69a (+competitor) probes using 30%^19^ and 25%^20^ formamide (FA), respectively. These FA concentrations were those optimized for the probes T0803-654 and HGC69a, although a lower FA concentration than that recommended for the probe CHL1851 of 35%^13^ was used. Samples mounted in Vectashield (Vectashield Laboratories, Burlingame, CA) were examined with an Olympus BX-51 epifluorescence microscope (Olympus, Tokyo, Japan).

### Statistical analysis

Stepwise multiple regression analysis was conducted to quantitatively examine how the environmental parameters in WWTP contributed to the copy number of *Kouleothrix* filamentous bacteria. The data for 12 environmental parameters except for SVI (Table 1) were collected from the municipal WWTP. However, as limited information on these 12 environmental parameters was available for some samples, only 22 sampling event data collected in 10/2016 (2 data sets), 1/2017 (1 data set), 3/2017 (4 data sets), 4/2017 (2 data sets), 5/2017 (2 data sets), 6/2017 (4 data sets), 7/2017 (3 data sets), 8/2017 (3 data sets), 9/2017 (1 data set) from those 42 samples were used for the analyses described here. The range and average of each parameter are shown in Table 1. Correlation analyses were performed for the 12 samples where amplicon sequencing data were generated (see Results) between the bacterial genera of interest and SVI values. All statistical analyses were performed with BellCurve for Excel (Social Survey Research Information Co., Ltd. Tokyo, Japan) with significant levels of 5% in the present study.

**Table 1:** Environmental parameter data for the A2O process train from 22 sludge samples used for stepwise multiple regression analysis.

	BOD^a^ [mg L^−1^]	T-N^a^ [mg L^−1^]	T-P^a^ [mg L^−1^]	SS^a^ [mg L^−1^]	SVI^b^ [mL g-sludge^−1^]	Temp.^b^ [°C]	pH^b^ [−]	DO^b^ [mg L^−1^]	MLSS^b^ [mg L^−1^]	SRT^b^ [d]	HRT^b^ [h]	Sludge return ratio^c^ [%]	SS in return sludge^c^ [mg L^−1^]
Range	44.0–101	14.2–30.1	1.47–4.20	17–44	115–405	16.4–27.5	6.5–6.8	1.7–6.4	1575–2560	5.85–19.5	6.58–8.45	50.0–75.6	4440–8270
Average	70.6	23.2	2.60	27	261	24.6	6.6	2.7	2144	12.8	7.53	53.7	6279
S.D.	17.2	4.23	7.00 × 10^−1^	6.8	88.7	3.46	9.2 × 10^−2^	1.0	308.0	3.82	5.46 × 10^−1^	8.07	1114

^a^Parameters in effluent water from the primary settlement tank (i.e., influent water into anaerobic tank).

^b^Parameters in aeration tank.

^c^Parameters in circulated return sludge from the secondary settlement tank to anaerobic tank.

## Results

### Identification of bulking bacteria in the A2O process train using amplicon sequencing, and their correlation with SVI

**Figure 1:**
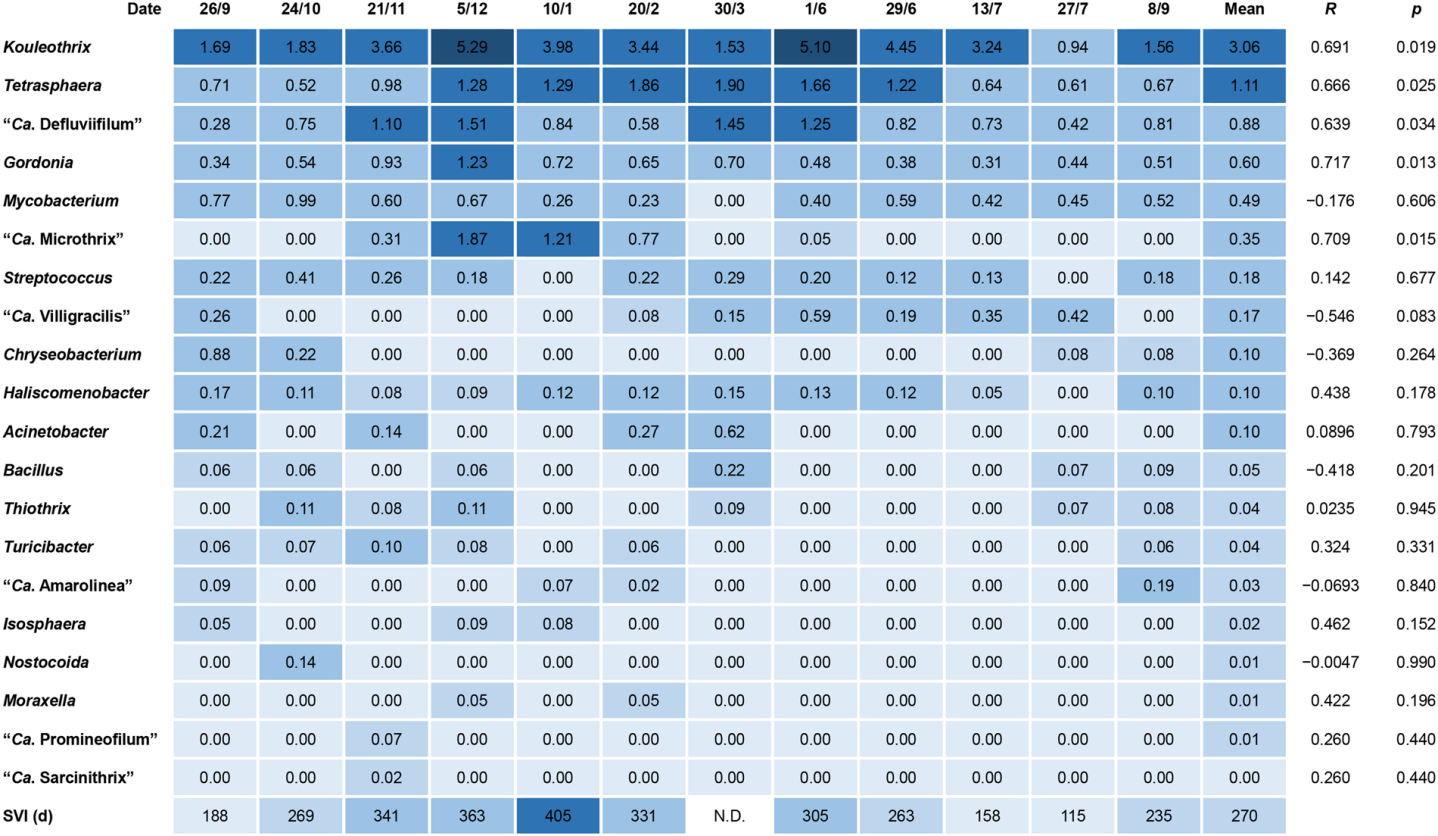
Heat map showing 16S rRNA gene copy number percentage abundances of putative filamentous bacteria in 12 activated sludge samples. *R* shows correlation coefficients between gene copy ratios and SVI.

Quantities of putative bulking bacteria in the 12 sludge samples after amplicon sequencing are expressed here as relative abundances of each as a percentage of the total number of amplicons. Figure 1 lists the filamentous genera mentioned in the MiDAS database and other published data^16,21–24^, although not all of the more abundant populations there should be considered as filamentous organisms. These data show that *Kouleothrix* was dominant, with a relative abundance of 3.06%, followed by *Tetrasphaera* sp., “*Ca*. Defluviifilum”, and *Gordonia* sp. with percentage relative abundances of 1.11%, 0.88%, and 0.60%, respectively. Figure 1 also shows the results of correlation analysis between SVI and these putative filaments. Only five populations: *Kouleothrix*, *Tetrasphaera* sp. “*Ca*. Defluviifilum”, *Gordonia* sp., and “*Ca*. Microthrix” correlated positively (*R* > 0.63) with statistical significance (*p* < 0.05).

**Figure 2:**
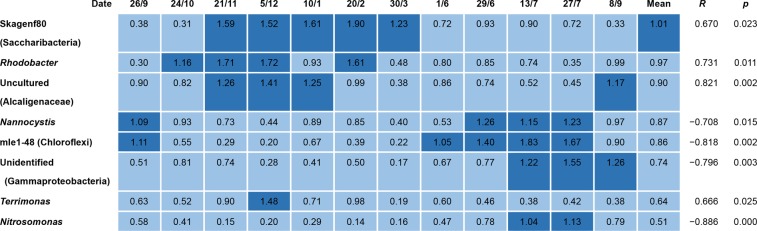
Heat map showing 16S rRNA gene copy percentage abundances of bacterial genera other than those from known putative filamentous bacteria given in Fig. 1, showing correlations with SVI in 12 activated sludge samples from the A2O process train. *R* shows correlation coefficients between gene copy ratio and SVI in Fig. 1.

To explore what impact the unidentified filamentous populations with amplicon percentage abundances >0.5% of the total abundances (Table S1) might have on SVI values in this community, correlation analysis was performed. Figure 2 shows four (Skagenf80 (“Ca. *Saccharibacteria*”), *Rhodobacter*, uncultured *Alcaligenaceae*, and *Terrimonas*) with positive and four (*Nannocystis*, strain mle1-48, unidentified *Gammaproteobacteria*, and *Nitrosomonas*) with negative correlations with SVI, at a statistically significant (*p* < 0.05) level. None of the four genera showing positive correlation has not been reported, possessing filamentous morphotype, but their individual relative mean abundances (0.64%−1.01%) might suggest that they may impact on sludge settleability. On the other hand, the other four genera with negative correlations may assist in improving sludge settleability. Although their individual relative abundances (0.51%–0.87%) were slightly lower than those non-filamentous populations showing a positive correlation, their correlations with SVI were more statistically significant. Thus, three of the four (strain mle1-48, unidentified *Gammaproteobacteria*, and *Nitrosomonas*) showed statistical significance (*p* < 0.01) with high correlation coefficients (<−0.796).

### FISH analysis of *Kouleothrix* present in activated sludge samples

**Figure 3:**
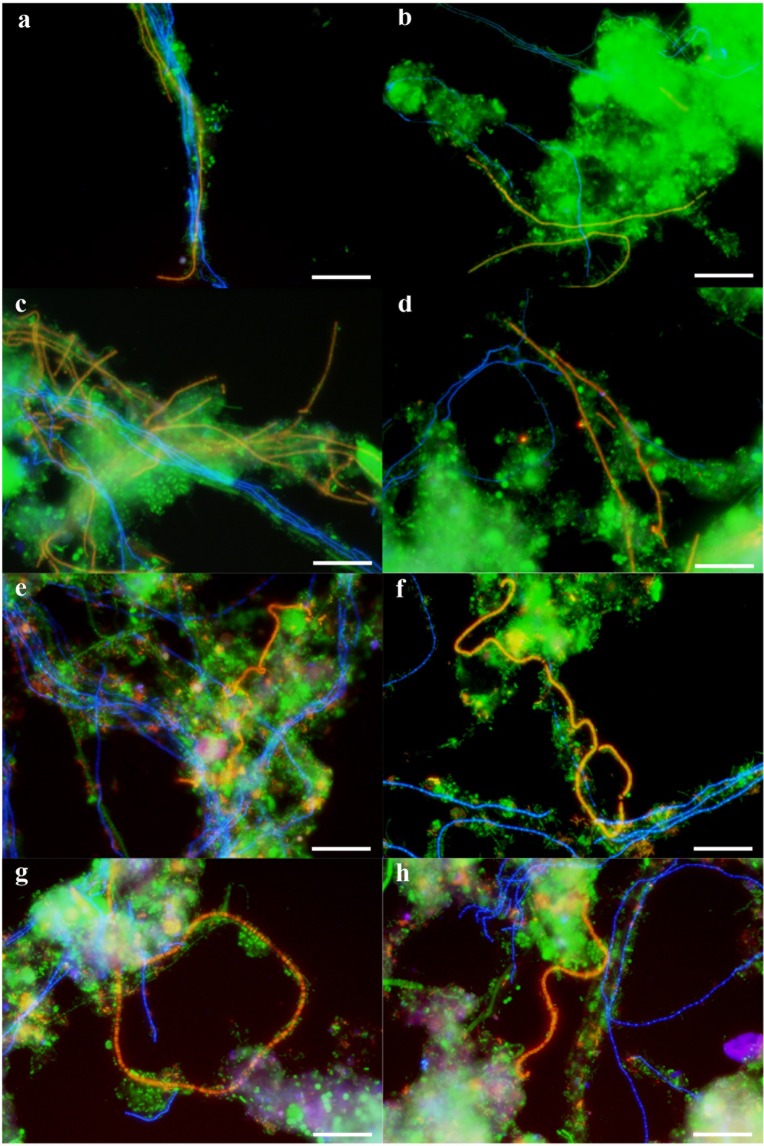
FISH composite images using probes EUBmix (Fluos, green), CHL1851 (Cy5, blue), and T0803-0654 (**a**–**d**) or HGC69a (**e**–**h**) (Cy3, red) on sludge samples (**a**,**e**) 5/12/2016, (**b**,**f**) 30/3/2017, (**c**,**g**) 1/6/2017, and (**d**,**h**) 27/7/2017. Probes CHL1851, T0803-0654, and HGC69a were designed to target *Kouleothrix* (type 1851), “*Ca*. Defluviifilum” (type 0803), and *Tetrasphaera* (*Nostocoida limicola* II), respectively. Yellow shows merged colors of green and red, and light blue also shows green and blue. Scale bars show 20 µm.

To confirm the morphology and location of *Kouleothrix* (type 1851), the most highly abundant population in the amplicon sequencing data, FISH analyses were performed on samples collected on 5/12/2016, 30/3/2017, 1/6/2017, and 27/7/2017. Figure 3a-d show FISH images of *Kouleothrix* with the third most abundant filament “*Ca*. Defluviifilum” (type 0803) (Fig. 1), where light blue and yellow images represent *Kouleothrix* and “*Ca*. Defluviifilum”, respectively. The samples show *Kouleothrix* filaments extending from the surface of flocs to form interfloc bridges (Fig. 3b–d), a pattern typical of bulking sludge^25^, and occasionally intertwining with those of “*Ca*. Defluviifilum” filaments (Fig. 3a). Figure 3e-h also show FISH images of *Kouleothrix* with the second most abundant filament *Tetrasphaera* (Fig. 1), readily recognizable as *T. jenkinsii* (*Nostocoida limicola* II) from its distinctive morphology of coiled and bent filaments with often irregular discoidal cells. Light blue and yellow images represent *Kouleothrix* and *T. jenkinsii*, respectively. All samples examined again showed *Kouleothrix* filaments extending from the surface of flocs, but not to extending around those of *T. jenkinsii*.

### Time course of increase and decrease in *Kouleothrix* 16S rRNA gene copy number and SVI

**Figure 4:**
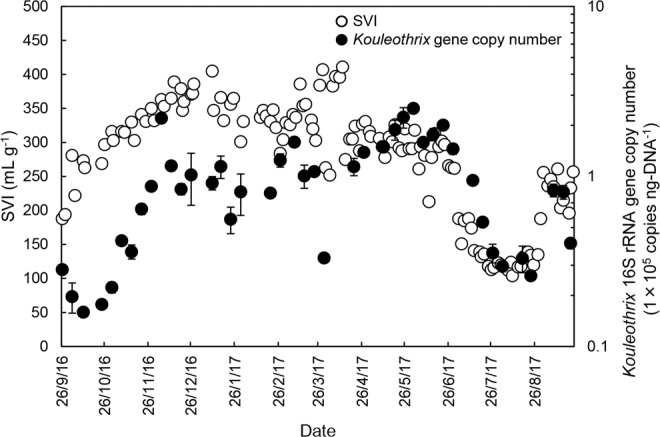
Time course of changes in *Kouleothrix* gene copy number and SVI. Error bar shows the standard deviation of *Kouleothrix* gene copy number (*n* = 3).

Forty-two sludge samples were collected from the A2O train during the one year period and further assayed specifically for *Kouleothrix* 16S rRNA gene copy number using real-time qPCR. The trends are shown in Fig. 4. The SVI values for the mixed liquors were provided by Yokohama City Government are also plotted (Fig. 4). The data show that *Kouleothrix* 16S rRNA gene copy number increased markedly from 10^4^ copies ng-DNA^−1^ to 10^5^ copies ng-DNA^−1^ between 9/2016 − 1/2017 and then gradually doubled in value between 1/2017–5/2017. From 6/2017, copy numbers decreased rapidly to around 10% of their previous value (i.e., 2.61 × 10^4^ copies ng-DNA^−1^) until 23/8/2017, when they then increased. As expected, SVI also rapidly increased from 188 to 405 mL g^−1^ between 9/2016 and 1/2017 and remained at >250 mL g^−1^ with frequent small oscillations from 1/2017 to 5/2017. The SVI fell to about 100 mL g^−1^ between 6/2017 and 23/8/2017 and then increased marginally. Thus, changes in the *Kouleothrix* 16S rRNA gene copy number mirrored changes in the biomass SVI over the whole monitoring period.

### Comparisons of amplicon sequencing data (*Kouleothrix* 16S rRNA gene abundance) and qPCR data (*Kouleothrix* 16S rRNA gene copy number)

**Figure 5:**
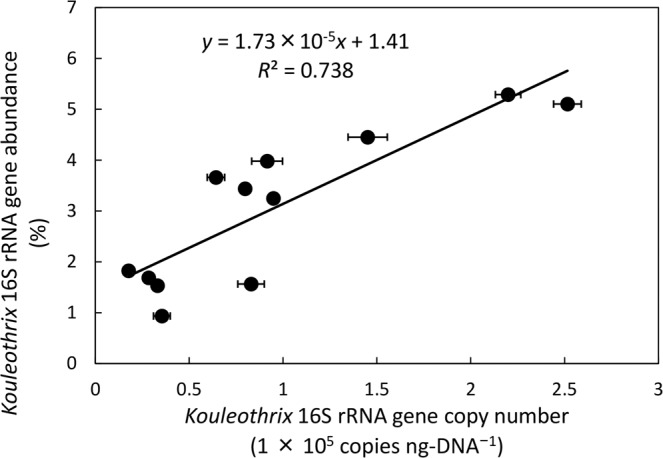
Relationship between *Kouleothrix* 16S rRNA gene copy number measured by qPCR and *Kouleothrix* 16S rRNA gene ratio determined by amplicon sequencing. Error bar shows the standard deviation of *Kouleothrix* gene copy number (*n* = 3).

To confirm the relationship between 16S rRNA gene copy numbers of *Kouleothrix* and its relative percentage abundances values generated by amplicon sequencing, comparisons between them were made (Fig. 5) for the following 12 sludge samples (26/9/2016, 24/10/2016, 21/11/2016, 5/12/2016, 10/1/2017, 20/2/2017, 30/3/2017, 1/6/2017, 29/6/2017, 13/7/2017, 27/7/2017, 8/9/2017) where amplicon sequence dates were available. The data show a positive statistically significant correlation between these two variables (*p* < 0.001) as follows (Eq. (2)):

1 $$y=1.73\times {10}^{-5}x+1.41$$

where *x* is *Kouleothrix* 16S rRNA gene copy number (copies ng-DNA^−1^) and *y* is *Kouleothrix* 16S rRNA gene relative abundances expressed as percentage of total number of bacterial 16S rRNA gene sequences.

### Multiple regression analysis of *Kouleothrix* 16S rRNA gene copy number and operational parameters

To explore which operational factors (Table 1) might affect *Kouleothrix* abundance, multiple regression analysis was conducted on 22 samples as described in the section “statistical analysis”. *Kouleothrix* 16S rRNA gene copy numbers and the following process operating parameters, SS, BOD, T-N, and T-P of effluent for the primary settling tank, mixed liquor temperature, pH, MLSS, DO, HRT, and SRT for the aeration tank, and sludge recirculation ratio, and return sludge SS were selected as dependent and independent variables, respectively. Stepwise multiple regression analysis revealed that increases in mixed liquor temperature negatively influenced *Kouleothrix* gene copy numbers, while SS, SRT, and return sludge SS variables each showed a positive correspondence (Table S2). The final four-variable model was statistically significant (see Table S3) and accounted for approximately 75% of the variance in the dependent variable (*R*^2^ = 0.75). The standardized *β*-coefficients for the measured parameters of temperature, SS, SRT, and return sludge SS were −0.712 (*p* < 0.01), 0.425 (*p* < 0.01), 0.568 (*p* < 0.01), and 0.524 (*p* < 0.01), respectively. These values clearly indicate that the four parameters contributed significantly to the regression model. The resulting multiple regression equation is as follows (Eq. (2)):

Predicted *Kouleothrix* gene copy number (copies ng−DNA^−1^) =

2 $$\begin{array}{cc} & -1.46\times {10}^{4}\,{\rm{m}}{\rm{i}}{\rm{x}}{\rm{e}}{\rm{d}}\,{\rm{l}}{\rm{i}}{\rm{q}}{\rm{u}}{\rm{o}}{\rm{r}}\,{\rm{t}}{\rm{e}}{\rm{m}}{\rm{p}}{\rm{e}}{\rm{r}}{\rm{a}}{\rm{t}}{\rm{u}}{\rm{r}}{\rm{e}}\,({}^{\circ }{\rm{C}})+4.46\times {10}^{3}\,{\rm{S}}{\rm{S}}\,({\rm{m}}{\rm{g}}\,{{\rm{L}}}^{-1})\\  & + 1.06\times {10}^{4}\,{\rm{S}}{\rm{R}}{\rm{T}}({\rm{d}})+\,33.5\,{\rm{r}}{\rm{e}}{\rm{t}}{\rm{u}}{\rm{r}}{\rm{n}}\,{\rm{s}}{\rm{l}}{\rm{u}}{\rm{d}}{\rm{g}}{\rm{e}}\,{\rm{S}}{\rm{S}}\,({\rm{m}}{\rm{g}}\,{{\rm{L}}}^{-1})-2.31\times {10}^{4}\end{array}$$

Table S2 also shows the variance inflation factor (VIF) values for temperature, SS, SRT, and return sludge SS, which were determined to be 2.20, 1.12, 1.84, and 1.64, respectively. All values were less than 10, indicating that there was no multicollinearity for these independent variables. These data as expected indicate that, increasing the SS, SRT, and return sludge SS values leads to an increase in the *Kouleothrix* 16 S rRNA gene copy number, which in turn negatively correlates with the mixed liquor temperature.

## Discussion

This present study aimed to investigate more closely the relationship between *Kouleothrix* filamentous bacteria abundances and SVI in a Japanese A2O process treating domestic wastewater. The qPCR data confirmed their important impact on sludge settleability in samples taken over 12 months. Yet Wágner *et al*. (2015)^26^ have questioned the role of the *Chloroflexi* in bulking. Although their FISH based study showed that their *Chloroflexi* were located predominately protruding from the floc surfaces, their modeling suggested they were less important than the floc bound actinobacterial “*Ca*. M. parvicella” in determining floc settlabilities. However, only phylum level CFX mix probes were used for *Chloroflexi* detection and quantification, and so no attempt was made to identify which individual *Chloroflexi* populations might be present in their community. This concern is especially applicable to *Kouleothrix* whose filament bundles act to join flocs together, as to whether either were present in the communities they describe.

This one-year investigation provides new insight into how plant operation parameters might favor the proliferation of *Kouleothrix* spp. and hence their role bulking. From multiple regression analysis, it was clear that the standardized *β*-coefficients for the mixed liquor temperature showed the highest absolute value of the four operational variables examined. Hence it seems unequivocal that *Kouleothrix* 16S rRNA gene copy numbers are affected by temperature, with higher percentage abundances detected at lower temperatures. The data were not included as a variable in the earlier study in generating the previous multiple regression^12^, since samples were taken mainly over summer no substantial temperature variations were recorded. As no previous studies have investigated a correlation between *Kouleothrix* growth rate in activated sludge and mixed liquor temperature, the data presented here show more meaningfully its response *in situ* than its behaviour in pure cultures. Optimal temperature for the growth of *Kouleothrix* isolates was reported as 25–30 °C by Kohno *et al*. (2002)^27^, which is higher than that ever recorded in the A2O plant used here, where the temperature ranged between 16.4 and 27.5 °C (Table 1). It appears *Kouleothrix* can replicate more rapidly at lower temperatures, but little relevant data are available to explain why and how. However, “*Ca*. M. parvicella” grows best at <20 °C in activated sludge systems^5,28^, while in pure cultures its maximum growth rate has been cited as >20 °C (22 °C^29^ and 25 °C^30^). It can still grow well at 7 °C according to Rossetti *et al*. (2002)^29^, leading them to suggest that this attribute would provide them with an advantage in cooler climates.

In the present study, both SS in influent and return sludge SS were also used as operational variables for multiple regression analysis (Eq. (2)). Both correlated with *Kouleothrix* relative percentage abundances. Mielczarec *et al*. (2012)^31^ have also reported a correlation between *Chloroflexi* type 1851 (i.e., *Kouleothrix*) and influent SS. How return sludge levels could lead to an increase in mixed liquor *Kouleothix* relative abundances is not easily interpreted, and could be seen as being counter-intuitive. Kragelund *et al*. (2007)^32^ using enzyme labelled fluorescence assays, could show presence of esterase activity for *Kouleothrix* isolate EU25 and *Kouleothrix* spp. in sludge flocs. Glucuronidase and galactosidase activities were also found. Their data would indicate that *Kouleothrix* spp. are well suited to degrade biopolymers, including complex polysaccharides. Kragelund et al. (2007)^32^ further hypothesized that they prefer to grow on colloidal particles present in the influent which had become trapped in the surrounding exopolymeric floc matrix, on exopolymers produced by other microbes, and on decaying bacterial cells. The statistical analysis in this and previous studies seem to be in accord with their physiological results. The proliferation of *Kouleothrix* spp. may be controllable, if the primary settling tank could be managed better to reduce SS levels in the influent.

Jenkins *et al*. (2004)^2^ in summarizing the earlier studies of Richard (1989)^33^ and Eikelboom (2000)^34^ where possible relationships between mean cell residence times (MCRT) and filamentous bacterial abundance were summarized, concluded that type 1851 filaments appeared in plants only at MCRT in excess of about 8 days. If so, it would appear that *Kouleothrix* spp. (type 1851) grows slowly *in situ*, regardless of temperature. The SRT range, average, and standard deviation (S.D.) of the A2O plant were 5.85–19.5 d, 12.8 d, and 3.82 d, respectively (Table 1), showing that the SRT was >8 days for most of the study period. Kohno *et al*. (2002)^27^ determined the *µ*_max_ of their *Kouleothrix* isolates during log phase, which was within the range 0.48 − 0.93 day^−1^. Whether *Kouleothrix* can grow in activated sludge at its *µ*_max_ is unlikely. However, the suggestion has been made that it might be possible to eliminate *Kouleothrix* bulking by reducing SRT^2,35^. The multiple regression equation generated in this present study (Eq. (2)) would support a method of wash out control, although we may also have to consider its impact on other functionally important slow growing populations such as the nitrifiers^36^.

Figure  4 shows the 16S rRNA gene copy numbers of *Kouleothrix* ranged from 1.59 × 10^4^ to 2.52 × 10^5^ copies ng-DNA^−1^. According to Eq. (1), the 16S rRNA gene ratio of *Kouleothrix* was estimated to be between 1.69% and 5.77% of the total copies. Earlier Nittami *et al*. (2017)^11^ discussed how copy number of *Kouleothrix* was associated with bulking, and compared their data with those obtained from similar studies targeting *Thiothrix eikelboomii*^7,8^. They used both qPCR and amplicon sequencing analyses to determine its 16S rRNA gene copy number and percentage relative abundance *in situ*, which showed 2.37 × 10^5^ copies ng-DNA^−1^ corresponded to 1.7% relative abundance of the total amplicons, respectively. When this *Kouleothrix* 16S rRNA gene copy number is inserted into Eq. (1), the relative percentage abundance of *Kouleothrix* emerges as 5.51%, which is almost triple that of the value obtained by Nittami *et al*. (2017)^11^ of 1.7%. In this present study, DNA extraction and the qPCR were performed under the optimized condition used previously^11^, while the amplicon sequencing analyses used different pipelines. Thus, the QIIME2 software package version 2019.01^15^ was used here, while in the earlier study^11^ used QIIME software package version 1.8.0^37^, the mean *Kouleothrix* relative percentage abundance was 1.98% which is lower than the 3.06% obtained here (Fig. 1). Furthermore, the PCR primers used here targeted the variable region (V3-4) while those used earlier^11^ targeted the V1-3 region. Albertsen *et al*. (2015)^38^ have reported how primer choice can influence markedly amplicon relative percentage abundances, where those of *Chloroflexi* using V3-4 primers (5.3%) were almost a third those generated by V1-3 primers (14%) and half those with the PCR-free method of Karst *et al*. (2018)^39^.

However, even taking this into account, and allowing for the absence largely of similar studies, *Kouleothrix* abundances seem higher than those determined in municipal WWTPs in other countries. For example, when Guo and Zhang (2012)^23^ looked at bulking and foaming (BFB) bacteria in 14 municipal (percentage of municipal wastewater 60–100%) activated sludge plants of China, Hong Kong, Singapore, Canada, and the United States targeting the V4 region, type 1851 was rarely found (totally 0.02%), although the most abundant BFB (*Nostocoida limicola* I (*Trichococcus*)) in those samples represented 2.06% of the total sequences. Again, primer bias may explain these low values^38^. When Nierychlo *et al*. (2018)^24^ described the distribution of *Chloroflexi* in 25 full-scale activated sludge plants in Denmark over 10 years, they too showed *Kouleothrix* spp. were present in low relative percentage abundances. Their amplicon sequence data agreed with their earlier qFISH results where *Kouleothrix* was present in all plants, but again always at low relative abundances (average 0.5% biovolume of total bacteria). Instead, four filaments “*Ca*. Defluviifilum”, “*Ca*. Promineofilum”, “*Ca*. Villigracilis”, and “*Ca*. Sarcinithrix” were the most abundant genera (all *Chloroflexi*), occupying 6.2% of the total reads in average across all the Danish plants.

Beer *et al*. (2002)^13^ showed with FISH that type 1851 filaments formed bundles of intertwined filaments, extending from activated sludge flocs, although Kragelund *et al*. (2007)^32^ claimed that their *Kouleothrix* were often seen as large bundles inside sludge flocs and were not always visible by phase contrast microscopy. Kragelund *et al*. (2011)^19^ also reported that type 0803 (now “*Ca*. Defluviifilum”) was located mainly at the surface of sludge flocs in most samples they examined. Here, both were seen both inside and outside of the flocs (Fig. 3) agreeing with the observations of Liao *et al*. (2004)^40^ who also suggested the ratio of extended filament length (measured as filament outside flocs) to total filament length (filaments inside and outside flocs) determined sludge settleability. This ratio was not determined here, although relative percentage abundances of both *Kouleothrix* and “*Ca*. Defluviifilum” clearly correlated with sludge SVI with statistical significance (Fig. 1).

The bacteria showing high correlation with SVI (Fig. 2) may also impact on sludge settlability. For example, some bacteria may support interfloc bridging and have a positive effect on settlability, even if they are non-filamentous. Further FISH analyses may reveal such an outcome. How some of these appear to improve floc settleability is not clear. For example, the *Nitrosomonas* (Fig. 2) may encourage denitrification in the anaerobic tank. However, *Kouleothrix* shows no denitrification ability^32^ and is less abundant in full scale nitrogen removal processes with anoxic tanks^12^.

In conclusion, this study has revealed that *Kouleothrix* (type 1851) causes the poor settleability of activated sludge biomass in full-scale WWTPs as a dominant filament. Statistical analyses of the microbial communities from a Japanese full-scale A2O plant over a one-year period has shown that changes in the operating parameters of temperature, SRT, SS, and return sludge SS may explain the dynamic behaviour of *Kouleothrix* spp. However, future work should be directed at manipulating each of these in a lab scale reactor and ultimately a full-scale treatment plant to confirm this relationship. Whole genome sequencing of cultured and uncultured *Kouleothrix* now underway, may reveal clues as to how it may be controlled.

## Supplementary information

Supplementary Information.

## Data Availability

The data that support the findings of this study are available from the corresponding author and/or Yokohama City Government upon reasonable request and with permission of the Government.
