# Supplementary material for: Exploring the operating factors controlling Kouleothrix (type 1851), the dominant filamentous bacterial population, in a full-scale A2O plant
Source: Sci Rep. 2020 Apr 22;10:6809. doi: 10.1038/s41598-020-63534-2 (PMC7176654; doi:10.1038/s41598-020-63534-2)
Supplement: Supplementary file 1 — Supplementary Information. [file 41598_2020_63534_MOESM1_ESM.pdf]

Exploring the operating factors controlling *Kouleothrix* (type 1851), the dominant filamentous bacterial population, in a full-scale A2O plant

Tadashi Nittami [1,*], Risa Kasakura [2], Toshimasa Kobayashi [2], Kota Suzuki [2], Yusuke Koshiba [1], Junji Fukuda [1], Minoru Takeda [1], Tomohiro Tobino [3, 4], Futoshi Kurisu [4, 5], Daniel Rice [6], Steve Petrovski [6], Robert J. Seviour [6]

[1] Division of Materials Science and Chemical Engineering, Faculty of Engineering, Yokohama National University, 79-5 Tokiwadai, Hodogaya-ku, Yokohama 240-8501, Japan

[2] Department of Chemistry, Chemical Engineering and Life Science, College of Engineering Science, Yokohama National University, 79-5 Tokiwadai, Hodogaya-ku, Yokohama 240-8501, Japan

[3] Department of Urban Engineering, Graduate School of Engineering, The University of Tokyo, 7-3-1 Hongo, Bunkyo-ku, Tokyo 113-8656, Japan

[4] Collaborative Research Institute for Innovative Microbiology, The University of Tokyo, 1-1-1 Yayoi, Bunkyo-ku, Tokyo 113-8657, Japan

[5] Research Center for Water Environment Technology, The University of Tokyo, 7-3-1 Hongo, Bunkyo-ku, Tokyo 113-8656, Japan

[6] Department of Physiology, Anatomy, and Microbiology, La Trobe University, Bundoora, VIC3086, Australia

Corresponding author at: Division of Materials Science and Chemical Engineering, Faculty of Engineering, Yokohama National University, 79-5 Tokiwadai, Hodogaya-ku, Yokohama 240-8501, Japan. Tel.: +81 45 339 4006, Fax: +81 45 339 4008.

E-mail address: nittami@ynu.ac.jp (T. Nittami).

Table S1 16S rRNA gene copy percentage abundances (%) of all bacterial genera detected in 12 A2O activated sludge samples.

	26/9	24/10	21/11	5/12	10/1	20/2	30/3	1/6	29/6	13/7	27/7	8/9	Mean
k__Bacteria;p__Nitrospirae;c__Nitrospira;o__Nitrospirales;f__Nitrospiraceae;g__Nitrospira;s__sublineage I	4.2812255	3.720084	3.91553	3.741782	2.457077	1.687318	0.129355	4.067083	4.006883	3.722371	2.698638	5.763274	3.349218
k__Bacteria;p__Proteobacteria;c__Betaproteobacteria;o__Rhodocyclales;f__Rhodocyclaceae;g__Zoogloea;s__	0.6534314	0.5646	0.587644	0.990233	3.510111	10.54679	14.16656	1.544677	1.457368	1.553398	1.36964	1.176254	3.176725
k__Bacteria;p__Chloroflexi;c__Chloroflexia;o__Chloroflexales;f__Roseiflexaceae;g__Kouleothrix;s__	1.6889238	1.825539	3.660989	5.289359	3.979397	3.438068	1.534413	5.102526	4.452873	3.247263	0.934769	1.563422	3.059795
k__Bacteria;p__Bacteroidetes;c__Sphingobacteriia;o__Sphingobacteriales;f__Chitinophagaceae;g__uncultured;s__	2.9886453	2.211348	1.109295	0.619572	2.125143	2.888316	7.734511	3.785307	3.150021	3.214212	3.198537	3.322271	3.028932
k__Bacteria;p__Bacteroidetes;c__Sphingobacteriia;o__Sphingobacteriales;f__Saprospiraceae;g__QEDR3BF09;s__	3.5599514	2.217622	2.372572	2.053516	3.643647	1.729606	3.28739	2.984112	3.35019	3.50754	3.926031	2.971976	2.967013
k__Bacteria;p__Proteobacteria;c__Deltaproteobacteria;o__Myxococcales;f__Haliangiaceae;g__Haliangium;s__	5.0417768	1.985509	1.709509	2.78131	2.445631	2.643041	0.677996	3.269283	3.680292	2.52014	3.100996	2.120206	2.664641
k__Bacteria;p__Bacteroidetes;c__Sphingobacteriia;o__Sphingobacteriales;f__Saprospiraceae;g__MK04;s__	2.7244162	2.245852	3.591855	3.254782	3.586417	1.979109	1.788661	1.92151	1.085124	1.31791	2.12152	4.188791	2.483829
k__Bacteria;p__Proteobacteria;c__Betaproteobacteria;o__Burkholderiales;f__Comamonadaceae;g__188up;s__	1.071199	2.578338	4.113506	3.606504	3.117131	1.966423	1.24002	2.311923	2.328276	2.710184	2.397887	2.039086	2.456706
k__Bacteria;p__Parcubacteria;c__;o__;f__;g__;s__	1.4782547	1.602836	1.021306	1.417711	1.987791	1.886074	3.688835	2.576725	1.510044	1.755836	3.617151	2.54056	2.09026
k__Bacteria;p__Saccharibacteria;c__;o__;f__;g__;s__	1.5853746	3.535021	2.224876	1.996699	1.335368	2.410454	2.011687	1.887561	1.562719	0.966742	1.158301	1.404867	1.839973
k__Bacteria;p__Proteobacteria;c__Betaproteobacteria;o__Rhodocyclales;f__Rhodocyclaceae;g__Candidatus Accumulibacter;s__	0.382061	0.439133	0.493369	0.148805	1.339184	1.911448	1.436282	3.37113	2.219413	3.491014	3.048161	2.673304	1.746109
k__Bacteria;p__Proteobacteria;c__Deltaproteobacteria;o__Myxococcales;f__mle1-27;g__mle1-27;s__	1.081911	1.646749	1.932625	2.407944	1.255246	1.37861	0	2.057306	2.665402	2.107003	3.048161	0.770649	1.695967
k__Bacteria;p__Bacteroidetes;c__Sphingobacteriia;o__Sphingobacteriales;f__Saprospiraceae;g__uncultured;s__	1.5211026	0.533233	0.593929	1.580044	1.686379	1.272889	0.735983	1.789109	1.074589	1.578186	1.52408	2.245575	1.344592
k__Bacteria;p__Proteobacteria;c__Betaproteobacteria;o__Rhodocyclales;f__Rhodocyclaceae;g__Dechloromonas;s__	0.403485	0.511276	0.323675	0.059522	1.308661	1.539307	3.006379	1.60239	1.552184	2.140054	1.743548	1.087758	1.273187
k__Bacteria;p__Bacteroidetes;c__Sphingobacteriia;o__Sphingobacteriales;f__Chitinophagaceae;g__Ferruginibacter;s__	2.1423981	1.634202	1.458111	1.942588	0.938573	1.129107	0.664615	0.818169	1.064054	1.152654	1.381833	0.722714	1.254085
k__Bacteria;p__Proteobacteria;c__Betaproteobacteria;o__Burkholderiales;f__Comamonadaceae;__;__	0.6962794	2.186255	2.215448	2.110333	1.18657	1.374382	0.753825	0.624661	0.965726	1.127866	0.699045	0.984513	1.243742
k__Bacteria;p__Proteobacteria;c__Gammaproteobacteria;o__Xanthomonadales;f__Xanthomonadaceae;g__Dokdonella;s__	2.1316861	1.709482	0.411665	0.229972	0.644792	0.871146	0.838574	1.877376	1.625931	1.479033	0.881935	1.40118	1.175231
k__Bacteria;p__Proteobacteria;c__Betaproteobacteria;o__Rhodocyclales;f__Rhodocyclaceae;g__uncultured;s__	1.0390631	1.794172	1.731507	1.512405	1.110263	0.718907	0.392524	0.967545	0.962214	1.160917	1.430604	1.183628	1.166979
k__Bacteria;p__Bacteroidetes;c__Sphingobacteriia;o__Sphingobacteriales;f__PHOS-HE51;g__;s__	1.0676284	1.455412	0.77305	1.041638	1.026326	1.936821	1.302467	1.144079	1.158871	0.987399	1.121723	0.837021	1.15437
k__Bacteria;p__Actinobacteria;c__Actinobacteria;o__Micrococcales;f__Intrasporangiaceae;g__Tetrasphaera;s__	0.7141327	0.51755	0.980454	1.282433	1.289584	1.860701	1.900174	1.663498	1.222082	0.640364	0.609632	0.667404	1.112334
k__Bacteria;p__Proteobacteria;c__Betaproteobacteria;o__Rhodocyclales;f__Rhodocyclaceae;g__Sulfuritalea;s__	0.6570021	1.201342	1.288417	0.603339	0.888974	0.824629	0.71368	1.446225	1.362551	1.371617	1.24365	1.25	1.070952
k__Bacteria;p__Saccharibacteria;c__Unknown Class;o__Unknown Order;f__SBR2060;g__;s__	0	0.919043	0.650493	0	9.576498	0	0.405906	0.129006	0	0.561867	0.150376	0.294985	1.057348
k__Bacteria;p__Bacteroidetes;c__Sphingobacteriia;o__Sphingobacteriales;f__Saprospiraceae;g__CYCU-0281;s__	0.8676712	0.699476	1.291559	0.941533	1.117894	0.693534	0.749364	0.882672	0.884956	1.028713	1.520016	1.725664	1.033588
k__Bacteria;p__Saccharibacteria;c__Unknown Class;o__FW73;f__S29;g__Skagenf80;s__	0.3784903	0.31053	1.586952	1.523227	1.613888	1.90299	1.231099	0.716323	0.93412	0.90064	0.719366	0.331858	1.012457
k__Bacteria;p__Proteobacteria;c__Alphaproteobacteria;o__Rhodobacterales;f__Rhodobacteraceae;g__Rhodobacter;s__	0.3035064	1.160566	1.709509	1.720733	0.930942	1.606969	0.477274	0.801195	0.85335	0.739517	0.345458	0.984513	0.969461
k__Bacteria;p__Bacteroidetes;c__Sphingobacteriia;o__Sphingobacteriales;f__env.OPS 17;g__;s__	1.0390631	0.476773	0.301678	0.156922	0.84319	1.425128	2.738748	1.049022	0.783116	0.760174	1.003861	0.601032	0.931559
k__Bacteria;p__Proteobacteria;c__Betaproteobacteria;o__Rhodocyclales;f__Rhodocyclaceae;__;__	1.3568521	1.292306	0.952171	0.714266	1.037772	1.243287	0.459432	0.519419	0.533783	0.702334	1.129852	0.877581	0.901588
k__Bacteria;p__Proteobacteria;c__Betaproteobacteria;o__Burkholderiales;f__Alcaligenaceae;g__uncultured;s__	0.8998072	0.821806	1.260134	1.409594	1.251431	0.985326	0.379143	0.862303	0.737463	0.516422	0.447064	1.165192	0.89464
k__Bacteria;p__Chloroflexi;c__Caldilineae;o__Caldilineales;f__Caldilineaceae;g__Candidatus Defluviifilum;s__	0.2820824	0.752799	1.10301	1.509699	0.839374	0.579355	1.449663	1.245926	0.818233	0.727122	0.41455	0.807522	0.877445
k__Bacteria;p__Proteobacteria;c__Deltaproteobacteria;o__Myxococcales;f__Nannocystaceae;g__Nannocystis;s__	1.0854817	0.925316	0.732198	0.435594	0.888974	0.845773	0.401445	0.526209	1.260711	1.148523	1.227393	0.969764	0.870615
k__Bacteria;p__Chloroflexi;c__TK10;o__mle1-48;f__mle1-48;g__mle1-48;s__	1.1069057	0.548916	0.285966	0.200211	0.671499	0.389056	0.223025	1.052417	1.404692	1.826069	1.674456	0.903392	0.857217
k__Bacteria;p__Proteobacteria;c__Alphaproteobacteria;o__Sphingomonadales;f__Sphingomonadaceae;__;__	1.0569164	1.160566	0.961599	1.415005	0.797406	0.490548	0.35684	0.400598	0.646158	0.702334	0.743751	0.60472	0.778037
k__Bacteria;p__Bacteroidetes;c__Cytophagia;o__Cytophagales;f__Cytophagaceae;g__uncultured;s__	1.1747483	0.586556	0.505939	0.284083	0.469287	0.452489	0.673536	1.524307	1.046495	0.95848	0.638082	0.671091	0.748758
k__Bacteria;p__Proteobacteria;c__Gammaproteobacteria;o__;f__;g__;s__	0.5106049	0.812396	0.73534	0.284083	0.408241	0.494777	0.17396	0.665399	0.77258	1.218756	1.55253	1.257375	0.740504
k__Bacteria;p__Nitrospirae;c__Nitrospira;o__Nitrospirales;f__Nitrospiraceae;g__Nitrospira;s__sublineage II	0.6355781	0.762209	1.407831	2.302427	0.682945	0.202986	0	0	0	0.144598	0.146312	2.14233	0.702268
k__Bacteria;p__Acidobacteria;c__Acidobacteria;o__Subgroup 4;f__Unknown Family;g__Blastocatella;s__	1.0890523	1.254666	0.590786	1.130922	0.572301	0.473633	0.312235	0.614476	0.481107	0.557736	0.605568	0.564159	0.68722
k__Bacteria;p__Proteobacteria;c__Alphaproteobacteria;o__Rhizobiales;f__A0839;g__;s__	0.5284582	1.251529	1.319842	0.868483	0.423502	0.469404	0.129355	0.750272	0.582947	0.470977	0.520219	0.814897	0.67749
k__Bacteria;p__Bacteroidetes;c__Sphingobacteriia;o__Sphingobacteriales;f__Chitinophagaceae;g__Terrimonas;s__	0.6248661	0.51755	0.901892	1.482644	0.709653	0.976868	0.191802	0.597501	0.456525	0.384218	0.41455	0.379794	0.636488
k__Bacteria;p__Actinobacteria;c__Actinobacteria;o__Corynebacteriales;f__Nocardiaceae;g__Gordonia;s__	0.3356424	0.539506	0.930174	1.231027	0.717283	0.651245	0.700299	0.482075	0.379267	0.309853	0.442999	0.512537	0.602659
k__Bacteria;p__Proteobacteria;c__Alphaproteobacteria;o__Sphingomonadales;f__Sphingomonadaceae;g__Sphingobium;s__	0.9283725	0.831216	0.38024	0.357133	0.702022	0.384827	0.869798	0.342884	0.64967	0.586656	0.735623	0.20649	0.581244
k__Bacteria;p__Chloroflexi;c__Caldilineae;o__Caldilineales;f__Caldilineaceae;g__uncultured;s__	1.1069057	0.31994	0.832757	0.909066	0.087753	0.190299	0.651233	0.553368	0.540806	1.086552	0.211339	0.328171	0.568183
k__Bacteria;p__Bacteroidetes;c__Flavobacteriia;o__Flavobacteriales;f__Flavobacteriaceae;g__Flavobacterium;s__	0.3677783	0.200747	1.112438	0.186683	0.427318	0.562439	2.631696	0.363254	0.228262	0.173518	0.256046	0.250737	0.56341
k__Bacteria;p__Bacteroidetes;c__Sphingobacteriia;o__Sphingobacteriales;f__AKYH767;g__;s__	0.4213383	0.41404	0.44309	0.565461	0.614269	1.129107	0.321156	0.855513	0.526759	0.508159	0.642146	0.291298	0.561028
k__Bacteria;p__Bacteroidetes;c__Flavobacteriia;o__Flavobacteriales;f__NS9 Marine group;g__PHOS-HE28;s__	0.6784261	1.028826	0.675633	0.286789	0.198398	0.456718	0.057987	0.716323	0.438966	0.739517	0.601504	0.553097	0.536015
k__Bacteria;p__Chloroflexi;c__Caldilineae;o__Caldilineales;f__Caldilineaceae;__;__	1.146183	0.771619	0.763623	0.530289	0.270889	0.287563	0.107052	0.750272	0.575924	0.243751	0.650274	0.210177	0.525635
k__Bacteria;p__Chlorobi;c__Chlorobia;o__Chlorobiales;f__OPB56;g__;s__	0.9783618	0.39522	0.370813	0.386894	0.446395	0.621643	0.579865	0.098452	0.526759	0.442057	0.857549	0.479351	0.51528
k__Bacteria;p__Proteobacteria;c__Betaproteobacteria;o__Nitrosomonadales;f__Nitrosomonadaceae;g__Nitrosomonas;s__	0.5748768	0.410903	0.150839	0.1948	0.289966	0.135324	0.156118	0.47189	0.783116	1.036976	1.133916	0.789086	0.510651
k__Bacteria;p__Actinobacteria;c__Actinobacteria;o__Corynebacteriales;f__Mycobacteriaceae;g__Mycobacterium;s__	0.7676926	0.994323	0.597071	0.670978	0.263258	0.232588	0	0.403992	0.589971	0.417269	0.447064	0.519912	0.49201
k__Bacteria;p__Proteobacteria;c__Betaproteobacteria;o__Burkholderiales;f__Comamonadaceae;g__Piscinibacter;s__	0.4606156	0.57401	0.763623	0.570872	0.362457	0.253732	0	0.427757	0.604017	0.681677	0.508027	0.689528	0.49136
k__Bacteria;p__Acidobacteria;c__Acidobacteria;o__Subgroup 4;f__11-24;g__;s__	0.8962365	0.19761	0.424235	0.424772	0.156429	0.118408	0.187341	0.712928	0.807698	0.756042	0.625889	0.545723	0.487776
k__Bacteria;p__Planctomycetes;c__OM190;o__;f__;g__;s__	0.328501	0	0.084847	0.100106	0.415872	0.380598	0.08475	0.960755	1.1378	1.127866	0.581183	0.630531	0.486067
k__Bacteria;p__Proteobacteria;c__Gammaproteobacteria;o__Xanthomonadales;f__Competibacteraceae;g__CPB_S60;s__	0.4284796	0.586556	0.725913	0.589811	0.209844	0.211443	0	0	0.586459	0.677546	0.613696	1.014012	0.470313
k__Bacteria;p__Proteobacteria;c__Betaproteobacteria;o__Burkholderiales;f__Comamonadaceae;g__spb280;s__	0.2106691	0.269753	0.449375	0.692622	0.888974	1.158709	0.820732	0.543183	0.361708	0.247883	0	0	0.470301
k__Bacteria;p__Verrucomicrobia;c__OPB35 soil group;o__;f__;g__;s__	0.4748982	0.30112	0.160266	0.211033	0.183136	0.308707	0.245328	0.709533	0.874421	0.656889	0.650274	0.630531	0.450511
k__Bacteria;p__Proteobacteria;c__Deltaproteobacteria;o__Myxococcales;f__Polyangiaceae;g__Byssovorax;s__	0.2035278	0.351306	0.512224	0.346311	0.495994	0.553981	0.419287	0.801195	0.498666	0.499897	0.284495	0.387168	0.446171
k__Bacteria;p__Elusimicrobia;c__Elusimicrobia;o__MVP-88;f__;g__;s__	0.403485	0.15056	0.072277	0	0.522701	0.114179	0	0.611081	0.309032	1.02045	1.154237	0.955015	0.442751
k__Bacteria;p__Spirochaetae;c__Spirochaetes;o__Spirochaetales;f__Leptospiraceae;g__Turneriella;s__	0.5213169	0.46109	0.373955	0.346311	0.423502	0.587812	0.129355	0.397203	0.54783	0.603181	0.495834	0.353982	0.436781
k__Bacteria;p__Proteobacteria;c__Alphaproteobacteria;o__Rhodobacterales;f__Rhodobacteraceae;g__Defluviimonas;s__	0.5570235	0.548916	0.587644	0.622278	0.50744	0.545524	0	0.251222	0.48813	0.301591	0.377972	0.427729	0.434622
k__Bacteria;p__Firmicutes;c__Clostridia;o__Clostridiales;f__Peptostreptococcaceae;g__Romboutsia;s__	0.6534314	0.316803	0.44309	0.695328	0.309042	0.397513	0.486195	0.366649	0.442478	0.297459	0.373908	0.339233	0.426761
k__Bacteria;p__Bacteroidetes;c__Sphingobacteriia;o__Sphingobacteriales;f__Saprospiraceae;g__Candidatus Epiflobacter;s__	0.392773	0.48932	0.348815	0.273261	0.358642	0.236817	0.08921	0.597501	0.632111	0.685809	0.536476	0.394543	0.419606
k__Bacteria;p__Proteobacteria;c__Betaproteobacteria;o__SC-I-84;__;__;__	0.2856531	0.58342	0.74791	0.735911	0.351011	0	0	0.186719	0.614553	0.636232	0.593375	0.283923	0.418226
k__Bacteria;p__Saccharibacteria;c__Unknown Class;o__FW73;__;__;__	0.2749411	0.483046	0.650493	0.527583	0.351011	0.042289	0.628931	0.302146	0.351173	0.504028	0.422678	0.34292	0.40677
k__Bacteria;p__Acidobacteria;c__Acidobacteria;o__Subgroup 17;f__CCU21;g__Btb7_22;s__	0.6534314	0.48932	0.411665	0.416655	0.244182	0.169155	0	0.213878	0.498666	0.669283	0.694981	0.379794	0.403417
k__Bacteria;p__Saccharibacteria;c__Unknown Class;o__FW73;f__S29;g__;s__	0.7784046	1.060193	0.153982	0.213739	0.118275	0.291792	0.276551	0.268197	0.375755	0.223094	0.59744	0.424041	0.398455
k__Bacteria;p__Proteobacteria;c__Deltaproteobacteria;o__Myxococcales;f__Polyangiaceae;g__Sorangium;s__	0.371349	0.853173	0.681918	0.762966	0.362457	0.397513	0	0.329305	0.361708	0.128073	0.219468	0.291298	0.396602
k__Bacteria;p__Chlorobi;c__Chlorobia;o__Chlorobiales;f__SJA-28;g__K2-30-37;s__	0.7355567	0.476773	0.44309	0.722383	0.133537	0.30025	0.133815	0.719718	0.382778	0.181781	0.052835	0.45354	0.394671
k__Bacteria;p__Chloroflexi;c__Anaerolineae;o__Anaerolineales;f__Anaerolineaceae;__;__	0.5034635	0.759073	0.82333	0.543816	0.236551	0.143781	0.080289	0.088267	0.16154	0.293328	0.300752	0.722714	0.388075
k__Bacteria;p__Proteobacteria;c__Gammaproteobacteria;o__Pseudomonadales;f__Moraxellaceae;g__uncultured;s__	0.8355352	0	0	0	0.553224	0.219901	0.633391	0.397203	0.572412	0.334642	0.451128	0.593658	0.382591
k__Bacteria;p__Chlorobi;c__Chlorobia;o__Chlorobiales;f__SJA-28;g__;s__	0.731986	0.57401	0.421092	0.792727	0.175506	0.219901	0.053526	0.15277	0.179098	0.256145	0.426743	0.409292	0.366066
k__Bacteria;p__Chloroflexi;c__Anaerolineae;o__Anaerolineales;f__Anaerolineaceae;g__B3-65;s__	0.2785117	0.379536	0.622211	0.538405	0.175506	0.122637	0	0.122216	0.828768	0.669283	0.382036	0.24705	0.363847
k__Bacteria;p__Proteobacteria;c__Deltaproteobacteria;o__Bdellovibrionales;f__Bdellovibrionaceae;g__Bdellovibrio;s__	0.8319646	0.269753	0.229401	0.124456	0.568485	0.312936	0.419287	0.353069	0.207192	0.194175	0.455192	0.35767	0.360298
k__Bacteria;p__Proteobacteria;c__Alphaproteobacteria;o__Sphingomonadales;f__Sphingomonadaceae;g__Zymomonas;s__	0.4856102	0.69634	0.449375	0.484294	0.183136	0.249503	0	0.247827	0.470572	0.313985	0.402357	0.309735	0.357728
k__Bacteria;p__Armatimonadetes;c__;o__;f__;g__;s__	0.339213	0.608513	0.427377	0.776494	0.312858	0.279105	0.486195	0.291961	0.179098	0.156992	0.215403	0.195428	0.35572
k__Bacteria;p__Actinobacteria;c__Acidimicrobiia;o__Acidimicrobiales;f__Microthricaceae;g__Candidatus Microthrix;s__	0	0	0.307963	1.872244	1.213277	0.765425	0	0.054318	0	0	0	0	0.351102
k__Bacteria;p__Planctomycetes;c__Phycisphaerae;o__Phycisphaerales;f__Phycisphaeraceae;g__SM1A02;s__	0.5605942	0.345033	0.059707	0	0.232736	0.503235	0.115973	0.370043	0.698834	0.553605	0.451128	0.276549	0.347286
k__Bacteria;p__Proteobacteria;c__Gammaproteobacteria;o__Xanthomonadales;f__Competibacteraceae;__;__	0	0.740253	0.883037	0.311139	0.274704	0	0	0.179929	0.586459	0.45032	0.321073	0.243363	0.332523
k__Bacteria;p__Proteobacteria;c__Betaproteobacteria;o__Burkholderiales;f__Comamonadaceae;g__Rhodoferax;s__	0.2785117	0.36699	0.637923	0.670978	0.351011	0.723136	0	0.190114	0.323079	0	0.195082	0.221239	0.329839
k__Bacteria;p__Firmicutes;c__Bacilli;o__Lactobacillales;f__Carnobacteriaceae;g__Trichococcus;s__	0.0856959	0.05646	0.273396	0.321961	0.534147	1.226371	1.0616	0.169745	0.073746	0.128073	0	0	0.3276
k__Bacteria;p__Actinobacteria;c__Actinobacteria;o__Bifidobacteriales;f__Bifidobacteriaceae;g__Bifidobacterium;s__	0.2463758	0.31053	0.421092	0.376072	0.339565	0.477862	0.593247	0.227458	0.242309	0.173518	0.191018	0.313422	0.326039
k__Bacteria;p__Bacteroidetes;c__Sphingobacteriia;o__Sphingobacteriales;__;__;__	0.2856531	0.43286	0.307963	0.186683	0.45021	0.18607	0.249788	0.281776	0.431943	0.470977	0.312945	0.309735	0.32555
k__Bacteria;p__WCHB1-60;c__;o__;f__;g__;s__	0.371349	0.26348	0.556219	0.530289	0.454025	0.109951	0	0	0.382778	0.35943	0.471449	0.324484	0.318621
k__Bacteria;p__Acidobacteria;c__Acidobacteria;o__Subgroup 6;f__mb2424;g__SBRFL126;s__	0.328501	0.48932	0.219974	0.21915	0.370088	0.173384	0.111513	0.298751	0.410872	0.409006	0.377972	0.368732	0.314772
k__Bacteria;__;__;__;__;__;__	0.3249304	0.04705	0.125699	0.5357	0.427318	0.219901	0.392524	0.220668	0.351173	0.35943	0.312945	0.39823	0.309631
k__Bacteria;p__Saccharibacteria;c__Unknown Class;o__Unknown Order;f__SBR2060;g__SBR2113;s__	0	0	0.45566	0.546522	0.362457	1.137565	0.454971	0.468495	0.133446	0	0.073156	0.077434	0.309142
k__Bacteria;p__Proteobacteria;c__Betaproteobacteria;o__Burkholderiales;f__Burkholderiaceae;g__Lautropia;s__	0.2856531	0.558326	0.386525	0.400422	0.293781	0.207214	0	0.353069	0.302009	0.26854	0.288559	0.243363	0.298955
k__Bacteria;p__Chloroflexi;c__Anaerolineae;o__Anaerolineales;f__Anaerolineaceae;g__uncultured;s__	0.4963222	0.398356	0.204261	0.411244	0.152614	0	0.330077	0.356464	0.375755	0.326379	0.272302	0.235988	0.296647
k__Bacteria;p__Actinobacteria;c__Actinobacteria;o__Propionibacteriales;f__Nocardioidaceae;g__Nocardioides;s__	0.1071199	0.439133	0.31739	0.321961	0.213659	0.14801	0.401445	0.373438	0.26338	0.305722	0.23166	0.427729	0.295887
k__Bacteria;p__Acidobacteria;c__Acidobacteria;o__Subgroup 4;f__DS-100;g__T1-5;s__	0.6784261	0.426586	0.223116	0.224561	0.106829	0.122637	0	0.027159	0.224751	0.487503	0.784393	0.235988	0.295163
k__Bacteria;p__Verrucomicrobia;c__Verrucomicrobiae;o__Verrucomicrobiales;f__Verrucomicrobiaceae;g__Prosthecobacter;s__	0.2713704	0.335623	0.103702	0.21915	0.488363	0.469404	0.396985	0.448126	0.256356	0	0.280431	0.213864	0.290281
k__Bacteria;p__Firmicutes;c__Clostridia;o__Clostridiales;f__Lachnospiraceae;g__Fusicatenibacter;s__	0.1499679	0.345033	0.273396	0.235383	0.202213	0.405971	0.811811	0.162955	0.270403	0.111547	0.174761	0.339233	0.290223
k__Bacteria;p__Actinobacteria;c__Actinobacteria;o__Propionibacteriales;f__Nocardioidaceae;g__Marmoricola;s__	0.2749411	0.699476	0.421092	0.397717	0.217474	0.105722	0	0.32591	0.249333	0.169386	0.317009	0.276549	0.287884
k__Bacteria;p__Proteobacteria;c__Betaproteobacteria;o__Burkholderiales;f__Comamonadaceae;g__Ideonella;s__	0	0.50814	0.461945	0.229972	0	0.37214	0	0.370043	0.316056	0.289196	0.455192	0.394543	0.283102
k__Bacteria;p__Proteobacteria;c__Epsilonproteobacteria;o__Campylobacterales;f__Campylobacteraceae;g__Arcobacter;s__	0.1535385	0.203883	0.119414	0.108222	0.274704	0.384827	1.217717	0.17314	0.154516	0	0.215403	0.387168	0.282711
k__Bacteria;p__Spirochaetae;c__Spirochaetes;o__Spirochaetales;f__Leptospiraceae;g__Leptospira;s__	0.2392344	0.05646	0.040852	0.054111	0.213659	0.676619	0.405906	0.074688	0.207192	0.760174	0.292623	0.324484	0.278833
k__Bacteria;p__Bacteroidetes;c__Sphingobacteriia;o__Sphingobacteriales;f__Chitinophagaceae;g__Parafilimonas;s__	0.4320503	0.49873	0.395952	0.427478	0.179321	0.152239	0	0.190114	0.323079	0.26854	0.239789	0.136431	0.27031
k__Bacteria;p__Bacteroidetes;c__Sphingobacteriia;o__Sphingobacteriales;f__KD3-93;g__;s__	0.2535171	0.313666	0.179121	0.167744	0.484548	0.435573	0.187341	0.207089	0.242309	0.247883	0.186954	0.235988	0.261811
k__Bacteria;p__Actinobacteria;c__Acidimicrobiia;o__Acidimicrobiales;f__Microthricaceae;g__P58;s__	0.3249304	0.627333	0.295393	0.140689	0.282335	0	0.169499	0.156165	0.287962	0.475108	0	0.368732	0.260679
k__Bacteria;p__Proteobacteria;c__Gammaproteobacteria;o__Cellvibrionales;f__Spongiibacteraceae;g__BD1-7 clade;s__	0.339213	0.106647	0.087989	0.032467	0.167875	0.194528	0.44159	0.295356	0.344149	0.363561	0.292623	0.431416	0.258118
k__Bacteria;p__Chloroflexi;c__Chloroflexia;o__Chloroflexales;f__Roseiflexaceae;__;__	0.1178319	0.16938	0.628496	0.489705	0.206028	0	0.156118	0.488865	0.196657	0.132204	0.134119	0.331858	0.254272
k__Bacteria;p__Proteobacteria;c__Gammaproteobacteria;o__Xanthomonadales;f__Xanthomonadales Incertae Sedis;g__Acidibacter;s__	0.2499464	0.457953	0.358243	0.094694	0.152614	0	0.124894	0.241037	0.354685	0.293328	0.268238	0.45354	0.254098
k__Bacteria;p__SHA-109;c__;o__;f__;g__;s__	0.1678212	0.203883	0.276538	0.311139	0.331934	0.363682	0	0.526209	0.26338	0.223094	0.016257	0.294985	0.248244
k__Bacteria;p__Proteobacteria;c__Gammaproteobacteria;o__Xanthomonadales;f__Xanthomonadaceae;g__sipK9;s__	0.4713276	0.109783	0	0	0	0.143781	0.26763	0.434546	0.175586	0.54121	0.288559	0.538348	0.247564
k__Bacteria;p__Gracilibacteria;c__;o__;f__;g__;s__	0.0607013	0.241523	0.040852	0.281378	0.244182	0.617414	0.495116	0.16635	0.16154	0.074365	0.284495	0.239676	0.242299
k__Bacteria;p__Proteobacteria;c__Gammaproteobacteria;o__Xanthomonadales;f__Competibacteraceae;g__Candidatus Contendobact	0.1463972	0.166243	0.157124	0.321961	0.099199	0	0.049066	0	0.407361	0.512291	0.715302	0.243363	0.234859
k__Bacteria;p__Proteobacteria;c__Betaproteobacteria;o__Nitrosomonadales;f__Nitrosomonadaceae;g__A0837;s__	0.4713276	0.2823	0.197976	0.127161	0.190767	0.266419	0.169499	0.190114	0.200169	0.338773	0.377972	0	0.234373
k__Bacteria;p__Proteobacteria;c__Gammaproteobacteria;o__Xanthomonadales;f__Xanthomonadaceae;g__B63;s__	0.2249518	0.100373	0	0.062228	0.370088	0.435573	0.521879	0.339489	0.200169	0.181781	0.101605	0.254425	0.232713
k__Bacteria;p__Proteobacteria;c__Alphaproteobacteria;o__Rhizobiales;f__Bradyrhizobiaceae;g__Bradyrhizobium;s__	0.4106263	0.25407	0.427377	0.275967	0	0.194528	0	0	0.259868	0.23962	0.312945	0.339233	0.226186
k__Bacteria;p__Proteobacteria;c__Betaproteobacteria;o__SC-I-84;f__A21b;g__A21b;s__	0.2249518	0.25407	0.44309	0.31655	0.099199	0	0	0.207089	0.172075	0.26854	0.30888	0.379794	0.222853
k__Bacteria;p__Proteobacteria;c__Alphaproteobacteria;o__Rhizobiales;f__Methylocystaceae;g__Methylocystis;s__	0.1892452	0.32935	0.477657	0.311139	0.34338	0	0	0.308935	0.235286	0	0.191018	0.261799	0.220651
k__Bacteria;p__Actinobacteria;c__Actinobacteria;o__PeM15;f__PeM15;g__;s__	0.0928372	0.5646	0.257683	0.105517	0	0	0.495116	0.210483	0.242309	0.161124	0.256046	0.224926	0.217553
k__Bacteria;p__Proteobacteria;c__Alphaproteobacteria;o__Caulobacterales;f__Caulobacteraceae;g__uncultured;s__	0.1606799	0.313666	0.373955	0.31655	0.267074	0.181841	0	0.190114	0.309032	0.218963	0.117862	0.140118	0.215821

k__Bacteria;p__Actinobacteria;c__Actinobacteria;o__Micrococcales;f__Intrasporangiaceae;__;__	0	0.373263	0.471372	0.408539	0	0	0	0.179929	0.442478	0.252014	0	0.442478	0.214173
k__Bacteria;p__Saccharibacteria;c__Unknown Class;o__FW73;f__;g__;s__	0.1463972	0	0	0.3896	0.629531	1.010699	0.124894	0	0	0	0	0	0.211887
k__Bacteria;p__Gemmatimonadetes;c__Gemmatimonadetes;o__Gemmatimonadales;f__Gemmatimonadaceae;g__Skagen138;s__	0	0.22584	0.204261	0.083872	0.267074	0.211443	0.334538	0.190114	0.168563	0.198306	0.325137	0.324484	0.211136
k__Bacteria;p__Actinobacteria;c__Acidimicrobiia;o__Acidimicrobiales;f__Microthrixaceae;g__ML817J-10;s__	0.1821038	0.332486	0.399095	0.265144	0	0	0	0.054318	0.291474	0.417269	0.284495	0.261799	0.207349
k__Bacteria;p__Cyanobacteria;c__Melainabacteria;o__Obscuribacterales;f__Obscuribacteraceae;g__Candidatus Obscuribacter;s__	0.2035278	0.15997	0.175979	0.081167	0.194582	0.202986	0.115973	0.32591	0.309032	0.260277	0.272302	0.147493	0.2041
k__Bacteria;p__Saccharibacteria;__;__;__;__;__	0.2392344	0.323076	0.279681	0.26785	0.22129	0	0.120434	0	0	0	0.690916	0.283923	0.2022
k__Bacteria;p__Planctomycetes;c__Planctomycetacia;o__Planctomycetales;f__Planctomycetaceae;g__Gemmata;s__	0.3570663	0.1882	0.138269	0.278672	0.068676	0	0.035684	0.298751	0.319567	0.289196	0.235724	0.20649	0.201358
k__Bacteria;p__Proteobacteria;c__Alphaproteobacteria;o__Caulobacterales;f__Hyphomonadaceae;g__Woodsholea;s__	0.339213	0.335623	0.179121	0.143394	0.198398	0	0.057987	0.176534	0.340638	0.289196	0.18289	0.169617	0.201051
k__Bacteria;p__Proteobacteria;c__Betaproteobacteria;o__Burkholderiales;f__Comamonadaceae;g__Acidovorax;s__	0.7141327	0.144287	0.276538	0.330078	0.206028	0.131095	0	0.101847	0	0.140467	0.174761	0.162242	0.198456
k__Bacteria;p__Verrucomicrobia;c__Opitutae;o__Opitutales;f__Opitutaceae;g__Opitutus;s__	0.3570663	0.097237	0	0	0.072491	0.228359	0.142736	0.363254	0.245821	0.334642	0.373908	0.162242	0.198146
k__Bacteria;p__Bacteroidetes;c__Sphingobacteriia;o__Sphingobacteriales;f__Chitinophagaceae;g__Niabella;s__	0	0	0	0.132572	0.354826	0.706221	0.79843	0.200299	0	0.152861	0	0	0.195434
k__Bacteria;p__Proteobacteria;c__Betaproteobacteria;o__Burkholderiales;f__Comamonadaceae;g__Ottowia;s__	0.1856745	0.297983	0.45566	0.173155	0.236551	0.325623	0	0.196904	0.16154	0	0.097541	0.20649	0.19476
k__Bacteria;p__Proteobacteria;c__Alphaproteobacteria;o__Sphingomonadales;f__Sphingomonadaceae;g__Novosphingobium;s__	0.1785332	0	0	0.289494	0.22892	0.418658	0.575405	0.4651	0	0.177649	0	0	0.19448
k__Bacteria;p__Bacteroidetes;c__Sphingobacteriia;o__Sphingobacteriales;f__Chitinophagaceae;g__Filimonas;s__	0.1928158	0.175653	0.188549	0.284083	0	0.507464	0	0	0.115887	0.297459	0.191018	0.368732	0.193472
k__Bacteria;p__Proteobacteria;c__Alphaproteobacteria;o__Rhodospirillales;f__B79;g__;s__	0.2035278	0.238386	0.204261	0.186683	0.22892	0.173384	0	0.14598	0.266891	0.309853	0.243853	0.114307	0.193004
k__Bacteria;p__Candidate division SR1;c__;o__;f__;g__;s__	0	0.1882	0.116272	0.083872	0.465471	0.452489	0.361301	0.30554	0.052676	0.074365	0.146312	0.062684	0.192432
k__Bacteria;p__Proteobacteria;c__Betaproteobacteria;o__Rhodocyclales;f__Rhodocyclaceae;g__Azospira;s__	0	0.335623	0.270253	0	0.144983	0.756967	0.312235	0.14598	0.193145	0.004131	0.113798	0.003687	0.190067
k__Bacteria;p__Proteobacteria;c__Gammaproteobacteria;o__Xanthomonadales;f__Competibacteraceae;g__CPB_P15;s__	0	0.116057	0.194834	0.181272	0.125906	0.101493	0	0.274986	0.393314	0.35943	0.317009	0.188053	0.187696
k__Bacteria;p__Proteobacteria;c__Gammaproteobacteria;o__Xanthomonadales;f__Competibacteraceae;g__Plasticicumulans;s__	0.2749411	0.17879	0.157124	0.064933	0.39298	0.181841	0.102592	0.074688	0	0.26854	0.227596	0.309735	0.186147
k__Bacteria;p__Proteobacteria;c__Gammaproteobacteria;o__Xanthomonadales;__;__;__	0.349925	0.410903	0.223116	0.132572	0.106829	0.046518	0	0.122216	0	0.260277	0.219468	0.34292	0.184562
k__Bacteria;p__Firmicutes;c__Bacilli;o__Lactobacillales;f__Streptococcaceae;g__Streptococcus;s__	0.2178105	0.40463	0.260826	0.175861	0	0.215672	0.294393	0.193145	0.115887	0.128073	0	0.180678	0.182561
k__Bacteria;p__Proteobacteria;c__Betaproteobacteria;o__Burkholderiales;f__Comamonadaceae;g__Variovorax;s__	0.2106691	0.232113	0.241971	0.202917	0.297596	0.084577	0.08475	0.169745	0.231774	0.194175	0.07722	0.143805	0.180943
k__Bacteria;p__Proteobacteria;c__Deltaproteobacteria;o__Myxococcales;f__Polyangiaceae;g__Polyangium;s__	0.2785117	0.12233	0	0.086578	0	0	0	0	0.238798	0.375955	0.853485	0.213864	0.180793
k__Bacteria;p__Actinobacteria;c__Acidimicrobiia;o__Acidimicrobiales;f__Microthrixaceae;g__;s__	0.2428051	0.29171	0.238828	0.175861	0	0.135324	0	0.224063	0.228262	0.218963	0.23166	0.176991	0.180372
k__Bacteria;p__Chloroflexi;c__Chloroflexia;o__Chloroflexales;f__Roseiflexaceae;g__;s__	0.2499464	0.26348	0.311106	0.3409	0.263258	0.152239	0.236407	0.115426	0.105352	0.115679	0	0	0.179483
k__Bacteria;p__Proteobacteria;c__Deltaproteobacteria;o__Myxococcales;f__Phaselicystidaceae;g__Phaselicystis;s__	0.2535171	0.12233	0.138269	0.143394	0	0.325623	0.124894	0.30554	0.193145	0.26854	0.166633	0.106932	0.179068
k__Bacteria;p__Bacteroidetes;c__Sphingobacteriia;o__Sphingobacteriales;f__NS11-12 marine group;g__;s__	0.05356	0.050187	0	0	0.160244	0.14801	1.266783	0.054318	0.08077	0.082628	0.065027	0.140118	0.175137
k__Bacteria;p__Actinobacteria;c__Acidimicrobiia;o__Acidimicrobiales;f__Iamiaceae;g__Iamia;s__	0	0.24466	0.436805	0.232678	0	0.253732	0	0.30554	0.266891	0	0.174761	0.180678	0.174645
k__Bacteria;p__Chloroflexi;c__Anaerolineae;o__Anaerolineales;f__Anaerolineaceae;g__Candidatus Villogracilis;s__	0.2642291	0	0	0	0	0.07612	0.147197	0.587317	0.189633	0.347036	0.418614	0	0.169179
k__Bacteria;p__Latescibacteria;c__;o__;f__;g__;s__	0.2606584	0.031367	0.094274	0.027056	0.106829	0	0	0.193509	0.323079	0.603181	0.357651	0	0.166467
k__Bacteria;p__Proteobacteria;c__Gammaproteobacteria;o__Xanthomonadales;f__uncultured;g__;s__	0.2106691	0.20702	0.201119	0.07305	0.072491	0.03806	0.066908	0	0.495154	0.202438	0.158504	0.269174	0.166216
k__Bacteria;p__Proteobacteria;c__Betaproteobacteria;o__Nitrosomonadales;f__Nitrosomonadaceae;g__uncultured;s__	0.2035278	0	0.084847	0.043289	0.183136	0.097264	0.249788	0.16635	0.252845	0.301591	0.223532	0.176991	0.165263
k__Bacteria;p__Bacteroidetes;__;__;__;__;__	0.3213597	0.994323	0.087989	0.062228	0.068676	0	0.035684	0	0.038629	0.045445	0	0.328171	0.165209
k__Bacteria;p__Hydrogenedentes;c__;o__;f__;g__;s__	0.2677998	0.222703	0.116272	0.17045	0.068676	0.139553	0.044605	0.071293	0.189633	0.231357	0.317009	0.132743	0.164341
k__Bacteria;p__Proteobacteria;c__Alphaproteobacteria;o__Rhizobiales;f__Hyphomicrobiaceae;g__Hyphomicrobium;s__	0	0	0	0.319255	0.160244	0.363682	0.191802	0.200299	0.207192	0.326379	0.150376	0	0.159936
k__Bacteria;p__Spirochaetae;c__Spirochaetes;o__Spirochaetales;f__Leptospiraceae;__;__	0.1356852	0.144287	0.103702	0.094694	0.156429	0.126866	0	0.295356	0.207192	0.177649	0.203211	0.239676	0.157062
k__Bacteria;p__OC31;c__;o__;f__;g__;s__	0.4534743	0	0.040852	0	0.125906	0.021144	0.071368	0.115426	0.210704	0.23962	0.15444	0.394543	0.15229
k__Bacteria;p__Acidobacteria;c__Acidobacteria;o__Subgroup 6;f__;g__;s__	0.2106691	0.116057	0.238828	0.17045	0.125906	0.160697	0.08921	0.200299	0.066723	0.070233	0.052835	0.324484	0.152199
k__Bacteria;p__Planctomycetes;c__Planctomycetacia;o__Planctomycetales;f__Planctomycetaceae;g__Planctomyces;s__	0.339213	0.26348	0.150839	0.135278	0	0.308707	0.156118	0.081477	0.119399	0.066102	0.162569	0.022124	0.150442
k__Bacteria;p__Bacteroidetes;c__Flavobacteriia;o__Flavobacteriales;f__Flavobacteriaceae;g__Cloacibacterium;s__	0.3677783	0.203883	0	0.086578	0.103014	0.122637	0.182881	0.122216	0.098328	0.165255	0.162569	0.173304	0.149037
k__Bacteria;p__Acidobacteria;c__Holophagae;o__Subgroup 10;f__ABS-19;g__Kaga01;s__	0.1928158	0.12233	0.185406	0.202917	0.068676	0	0	0.142586	0.231774	0.206569	0.211339	0.217552	0.148497
k__Bacteria;p__Proteobacteria;c__Alphaproteobacteria;o__Rhodospirillales;f__Acetobacteraceae;__;__	0	0.144287	0	0.235383	0.324304	0.143781	0.298854	0.288566	0	0.23962	0	0	0.139566
k__Bacteria;p__Proteobacteria;c__Deltaproteobacteria;o__Oligoflexales;f__;g__;s__	0.1249732	0.266616	0.094274	0	0.076307	0.241045	0.316696	0.057713	0.154516	0.09089	0.052835	0.165929	0.136816
k__Bacteria;p__Gemmatimonadetes;c__Gemmatimonadetes;o__Gemmatimonadales;f__Gemmatimonadaceae;g__C1711WL;s__	0.4249089	0.072143	0	0.051406	0.167875	0	0	0	0.105352	0	0.377972	0.431416	0.135923
k__Bacteria;p__Cyanobacteria;c__Melainabacteria;o__Obscuribacterales;f__Obscuribacteraceae;g__;s__	0.2785117	0.21643	0.40538	0.300317	0	0	0	0	0	0.111547	0.166633	0.125369	0.133682
k__Bacteria;p__Proteobacteria;c__Alphaproteobacteria;o__Rhodospirillales;f__I-10;g__;s__	0.2606584	0.241523	0.263968	0.202917	0	0	0	0.084872	0.10184	0.194175	0.195082	0.047935	0.132748
k__Bacteria;p__Proteobacteria;c__TA18;o__;f__;g__;s__	0.1713918	0.22584	0.087989	0.02435	0.091568	0	0.053526	0.105242	0.235286	0.293328	0.243853	0.051622	0.132
k__Bacteria;p__Bacteroidetes;c__Cytophagia;o__Cytophagales;f__Cytophagaceae;__;__	0.0249946	0.050187	0.056565	0	0.152614	0.211443	0.245328	0.071293	0.186122	0.247883	0.195082	0.125369	0.130573
k__Bacteria;p__Proteobacteria;c__Deltaproteobacteria;o__Oligoflexales;f__Oligoflexaceae;g__;s__	0.2035278	0.228977	0	0	0.030523	0	0.040145	0.030554	0.140469	0.38835	0.341394	0.154867	0.1299
k__Bacteria;p__Chloroflexi;c__WCHB1-50;o__WCHB1-50;f__WCHB1-50;g__WCHB1-50;s__	0.1035492	0.097237	0.160266	0.386894	0.106829	0.114179	0	0.17314	0.168563	0.09089	0.065027	0.077434	0.128667
k__Bacteria;p__Proteobacteria;c__Betaproteobacteria;__;__;__;__	0.2499464	0	0.003142	0.056817	0.16406	0.181841	0.124894	0.274986	0	0.289196	0.195082	0	0.12833
k__Bacteria;p__Proteobacteria;c__Betaproteobacteria;o__Hydrogenophilales;f__Hydrogenophilaceae;g__uncultured;s__	0.0571306	0.106647	0	0	0	0.029602	0.075829	0.342884	0.266891	0.2107	0.321073	0.103245	0.126167
k__Bacteria;p__Actinobacteria;c__Actinobacteria;o__Kineosporiales;f__Kineosporiaceae;g__Quadrisphaera;s__	0	0.232113	0.226259	0.202917	0	0	0	0.227458	0.231774	0.123941	0.10567	0.162242	0.126031
k__Bacteria;p__Proteobacteria;c__Betaproteobacteria;o__SC-I-84;f__;g__;s__	0	0.17879	0.279681	0.427478	0.175506	0.03806	0	0	0.08077	0.23962	0	0.044248	0.122013
k__Bacteria;p__Proteobacteria;c__Betaproteobacteria;o__Rhodocyclales;f__Rhodocyclaceae;g__Denitratisoma;s__	0.010712	0.109783	0.003142	0	0	0.266419	0.477274	0	0.18261	0	0.227596	0.077434	0.112914
k__Bacteria;p__Proteobacteria;c__Deltaproteobacteria;o__Bdellovibrionales;f__Bdellovibrionaceae;g__OM27 clade;s__	0.2070985	0.04705	0	0	0.175506	0.152239	0.151657	0.122216	0	0.066102	0.174761	0.250737	0.112281
k__Bacteria;p__Proteobacteria;c__Alphaproteobacteria;o__Rhodospirillales;f__Acetobacteraceae;g__uncultured;s__	0.1142612	0.247796	0.081704	0.159628	0.103014	0.093035	0	0.112032	0.210704	0.078496	0	0.143805	0.11204
k__Bacteria;p__Proteobacteria;c__Alphaproteobacteria;o__Rickettsiales;f__Rickettsiaceae;g__uncultured;s__	0.0892666	0.109783	0.069135	0.102811	0.05723	0.131095	0.26317	0.054318	0.087793	0.14873	0.174761	0.051622	0.111643
k__Bacteria;p__Proteobacteria;c__Deltaproteobacteria;o__Myxococcales;f__Sandaracinaceae;g__uncultured;s__	0.1606799	0.040777	0.040852	0.054111	0.072491	0.164926	0	0.339489	0.084282	0.107416	0.26011	0	0.110428
k__Bacteria;p__Actinobacteria;c__Acidimicrobiia;o__Acidimicrobiales;f__Acidimicrobiaceae;g__CL500-29 marine group;s__	0	0.200747	0.43052	0.192094	0	0	0.182881	0.179929	0	0.111547	0	0	0.108143
k__Bacteria;p__Proteobacteria;c__Deltaproteobacteria;o__Myxococcales;f__P3OB-42;g__;s__	0.1321145	0.043913	0.06285	0.062228	0.072491	0.131095	0.102592	0.247827	0.175586	0.103284	0.07722	0.077434	0.107386
k__Bacteria;p__Proteobacteria;c__Alphaproteobacteria;o__Rhizobiales;f__Nordellaceae;g__MNG7;s__	0	0.32935	0.125699	0	0	0	0	0.186719	0.158028	0.218963	0	0.254425	0.106099
k__Bacteria;p__Firmicutes;c__Clostridia;o__Clostridiales;f__Clostridiaceae 1;g__Clostridium sensu stricto 1;s__	0.1499679	0.0941	0.153982	0.1461	0	0.084577	0	0.156165	0.129934	0.152861	0.125991	0.077434	0.105926
k__Bacteria;p__Bacteroidetes;c__Sphingobacteriia;o__Sphingobacteriales;f__Saprospiraceae;g__Lewinella;s__	0.042848	0	0	0.186683	0.198398	0.228359	0.392524	0.17314	0.045652	0	0	0	0.105634
k__Bacteria;p__Bacteroidetes;c__Flavobacteriia;o__Flavobacteriales;f__Flavobacteriaceae;g__Chryseobacterium;s__	0.8748125	0.21643	0	0	0	0	0	0	0	0	0.07722	0.081121	0.104132
k__Bacteria;p__Bacteroidetes;c__Sphingobacteriia;o__Sphingobacteriales;f__Saprospiraceae;g__Haliscomenobacter;s__	0.1678212	0.11292	0.078562	0.091989	0.118275	0.122637	0.147197	0.125611	0.122911	0.053708	0	0.099558	0.103432
k__Bacteria;p__Proteobacteria;c__Deltaproteobacteria;o__Desulfarculales;f__Desulfarculaceae;g__uncultured;s__	0.0499893	0	0	0	0	0	0.022303	0.132401	0.424919	0.363561	0.158504	0.081121	0.102733
k__Bacteria;p__Proteobacteria;c__Gammaproteobacteria;o__Pseudomonadales;f__Moraxellaceae;g__Acinetobacter;s__	0.2142398	0	0.135127	0	0	0.266419	0.615549	0	0	0	0	0	0.102611
k__Bacteria;p__Proteobacteria;c__Deltaproteobacteria;o__GR-WP33-30;f__;g__;s__	0.2285225	0.128603	0.153982	0.143394	0.05723	0.029602	0	0.098452	0.098328	0.086759	0.097541	0.084808	0.100602
k__Bacteria;p__Proteobacteria;c__Alphaproteobacteria;o__Rhodospirillales;f__Acetobacteraceae;g__Stella;s__	0.1071199	0.109783	0.150839	0.075756	0	0.042289	0	0.210483	0.098328	0.09089	0.166633	0.066372	0.100004
k__Bacteria;p__Proteobacteria;c__Deltaproteobacteria;o__Desulfuromonadales;f__GR-WP33-58;g__32C6;s__	0.1285439	0.15056	0.116272	0.124456	0.072491	0.084577	0.124894	0	0.136957	0.107416	0.048771	0.099558	0.099541
k__Bacteria;p__Planctomycetes;c__Planctomycetacia;o__Planctomycetales;f__Planctomycetaceae;g__uncultured;s__	0.2356638	0.228977	0.094274	0.081167	0	0	0.08921	0.095057	0	0.095022	0.032514	0.224926	0.098068
k__Bacteria;p__Lentisphaerae;c__PBS-III-20;o__;f__;g__;s__	0.2106691	0.138013	0.144554	0	0	0.080348	0	0.129006	0.175586	0.128073	0.093477	0.066372	0.097175
k__Bacteria;p__Proteobacteria;c__Deltaproteobacteria;o__Myxococcales;f__uncultured;g__;s__	0.1713918	0.081553	0.02514	0.059522	0.156429	0	0	0.064503	0.108864	0.194175	0.142248	0.132743	0.094714
k__Bacteria;p__Acidobacteria;c__Acidobacteria;o__Subgroup 3;f__Elev-16S-1166;g__;s__	0	0.059597	0.179121	0.108222	0	0	0	0.302146	0.238798	0	0.243853	0	0.094311
k__Bacteria;p__Firmicutes;c__Bacilli;o__Lactobacillales;f__Streptococcaceae;g__Lactococcus;s__	0	0	0	0.167744	0.27852	0.131095	0.553102	0	0	0	0	0	0.094205
k__Bacteria;p__Proteobacteria;c__Alphaproteobacteria;o__Rhodospirillales;f__Rhodospirillaceae;g__uncultured;s__	0.2356638	0.505003	0.197976	0.0487	0.011446	0	0	0.067898	0	0.061971	0	0	0.094055
k__Bacteria;p__Proteobacteria;c__Alphaproteobacteria;o__Rhodobacterales;f__Rhodobacteraceae;g__Pseudorhodobacter;s__	0	0	0.135127	0.200211	0.289966	0.380598	0	0	0.10184	0	0	0	0.092312
k__Bacteria;p__Acidobacteria;c__Acidobacteria;o__Subgroup 3;f__Unknown Family;g__Bryobacter;s__	0.2035278	0.050187	0.100559	0.208328	0	0.33408	0	0	0	0	0	0.20649	0.091931
k__Bacteria;p__Proteobacteria;c__Alphaproteobacteria;o__Rhodospirillales;f__Rhodospirillaceae;g__Dongia;s__	0	0	0	0.0487	0.099199	0	0	0.071293	0.210704	0.293328	0.219468	0.158555	0.09177
k__Bacteria;p__Actinobacteria;c__Acidimicrobiia;o__Acidimicrobiales;f__uncultured;g__;s__	0.0928372	0.040777	0.285966	0.257028	0	0	0.115973	0	0.105352	0.095022	0.052835	0.05531	0.091758
k__Bacteria;p__Chloroflexi;c__Thermomicrobia;o__JG30-KF-CM45;f__;g__;s__	0	0	0.238828	0.21915	0.103014	0	0.147197	0.105242	0.108864	0	0	0.173304	0.0913
k__Bacteria;p__Proteobacteria;c__Betaproteobacteria;o__B1-7BS;f__;g__;s__	0	0.203883	0.106844	0	0	0	0.129355	0	0.277427	0	0	0.346608	0.088676
k__Bacteria;p__Proteobacteria;c__Gammaproteobacteria;o__Xanthomonadales;f__Xanthomonadaceae;g__Thermomonas;s__	0	0	0	0	0	0	0.307775	0.31233	0.242309	0.095022	0	0.092183	0.087468
k__Bacteria;p__Proteobacteria;c__Alphaproteobacteria;o__Rhizobiales;f__Rhizobiales Incertae Sedis;g__Rhizomicrobium;s__	0	0	0	0.113633	0.129721	0.342538	0.182881	0.122216	0.151004	0	0	0	0.086833
k__Bacteria;p__Proteobacteria;c__Deltaproteobacteria;o__Myxococcales;f__Sandaracinaceae;g__Sandaracinus;s__	0.0856959	0.081553	0.094274	0	0	0	0	0.139191	0.165051	0.2107	0.15444	0.110619	0.086794
k__Bacteria;p__Actinobacteria;c__Thermoleophilia;o__Solirubrobacterales;__;__;__	0	0.156833	0.056565	0.108222	0	0.080348	0	0.15956	0.119399	0.115679	0.089413	0.154867	0.08674
k__Bacteria;p__Proteobacteria;c__Gammaproteobacteria;o__Oceanospirillales;f__Halomonadaceae;g__Candidatus Accumulimonas;s_	0	0	0	0	0.125906	0.063433	0.361301	0.183324	0.175586	0	0.073156	0.044248	0.08558
k__Bacteria;p__Bacteroidetes;c__Cytophagia;o__Cytophagales;f__Cytophagaceae;g__;s__	0.042848	0.04705	0.06285	0.100106	0.103014	0.253732	0.093671	0.064503	0.042141	0.078496	0.093477	0.044248	0.085511
k__Bacteria;p__TM6;c__;o__;f__;g__;s__	0.0749839	0.138013	0.069135	0	0	0.03806	0.133815	0.213878	0.052676	0.103284	0.097541	0.103245	0.085386
k__Bacteria;p__Proteobacteria;c__Alphaproteobacteria;o__Rhizobiales;f__Hyphomicrobiaceae;g__Devosia;s__	0	0.153697	0	0.129867	0.110645	0.152239	0.200723	0.169745	0.087793	0	0	0	0.083726
k__Bacteria;p__Proteobacteria;c__Alphaproteobacteria;o__Sphingomonadales;f__Sphingomonadales Incertae Sedis;g__M05-Pitesti;s_	0.1249732	0.166243	0.109987	0.1461	0.137352	0	0	0	0	0	0.138183	0.180678	0.083626
k__Bacteria;p__Proteobacteria;c__Alphaproteobacteria;o__Rhodospirillales;f__Rhodospirillales Incertae Sedis;g__Candidatus Alysiospl	0	0.238386	0.204261	0.232678	0	0.173384	0.147197	0	0	0	0	0	0.082992
k__Bacteria;p__Proteobacteria;c__Deltaproteobacteria;o__Myxococcales;f__Blrii41;g__;s__	0.1535385	0.36699	0.160266	0.059522	0	0	0	0	0	0	0	0.254425	0.082895
k__Bacteria;p__Proteobacteria;c__Betaproteobacteria;o__Nitrosomonadales;f__Nitrosomonadaceae;__;__	0	0	0.282823	0.135278	0.095383	0	0	0	0.16154	0	0	0.317109	0.082678
k__Bacteria;p__Actinobacteria;c__Thermoleophilia;o__Solirubrobacterales;f__480-2;g__K2-78;s__	0.0999786	0.181927	0.097417	0.064933	0.061045	0	0	0	0.136957	0.111547	0.097541	0.129056	0.0817
k__Bacteria;p__Verrucomicrobia;c__Verrucomicrobiae;o__Verrucomicrobiales;f__Verrucomicrobiaceae;g__Verrucomicrobium;s__	0.0285653	0	0.273396	0	0	0.228359	0.080289	0.040739	0.119399	0	0.142248	0.051622	0.080385
k__Bacteria;p__Proteobacteria;c__Alphaproteobacteria;o__Caulobacterales;f__Caulobacteraceae;__;__	0	0	0.109987	0	0.22129	0.287563	0.338998	0	0	0	0	0	0.07982
k__Bacteria;p__Proteobacteria;c__Alphaproteobacteria;o__Rhodospirillales;f__Rhodospirillales Incertae Sedis;g__Reyranella;s__	0.2142398	0.222703	0.07542	0.1461	0	0	0	0	0	0.070233	0.097541	0.099558	0.07715
k__Bacteria;p__Bacteroidetes;c__Sphingobacteriia;o__Sphingobacteriales;f__Chitinophagaceae;__;__	0.0571306	0	0	0.119044	0	0.097264	0.553102	0.091662	0	0	0	0	0.076517
k__Bacteria;p__Proteobacteria;c__Betaproteobacteria;o__Burkholderiales;f__Comamonadaceae;g__Simplicispira;s__	0	0	0	0.083872	0.003815	0.418658	0.410366	0	0	0	0	0	0.076393
k__Bacteria;p__Planctomycetes;c__Phycisphaerae;o__Phycisphaerales;f__Phycisphaeraceae;g__CL500-3;s__	0.05356	0.153697	0.047137	0.032467	0	0	0	0	0.18261	0.214832	0.085349	0.136431	0.075507
k__Bacteria;p__Planctomycetes;c__Planctomycetacia;o__Planctomycetales;f__Planctomycetaceae;g__Pirellula;s__	0.1642505	0.050187	0	0	0.095383	0.177612	0	0.047529	0.094817	0.061971	0.18289	0.025811	0.075037
k__Bacteria;p__Firmicutes;c__Clostridia;o__Clostridiales;f__Ruminococcaceae;g__Subdoligranulum;s__	0.1071199	0	0.116272	0.156922	0.17169	0	0.075829	0.132401	0	0	0	0.132743	0.074415
k__Bacteria;p__Proteobacteria;c__Deltaproteobacteria;o__Desulfuromonadales;f__GR-WP33-58;g__;s__	0.1463972	0	0	0	0.137352	0.046518	0	0.132401	0.175586	0	0	0.228614	0.072239
k__Bacteria;p__Proteobacteria;c__Alphaproteobacteria;o__Rhizobiales;f__Phyllobacteriaceae;g__Nitratireductor;s__	0	0	0.204261	0	0	0	0.294393	0	0.108864	0	0	0.232301	0.069985
k__Bacteria;p__Proteobacteria;c__Gammaproteobacteria;o__Xanthomonadales;f__Xanthomonadaceae;g__Lysobacter;s__	0	0	0.82333	0	0	0	0	0	0	0	0	0	0.068611
k__Bacteria;p__Proteobacteria;c__Alphaproteobacteria;o__DB1-14;f__;g__;s__	0.1106906	0.119193	0	0	0.041969	0	0.107052	0.037344	0.042141	0.128073	0.158504	0.062684	0.067304
k__Bacteria;p__Bacteroidetes;c__Cytophagia;o__Cytophagales;__;__;__	0.0499893	0.040777	0	0.040583	0.064861	0	0.062447	0	0.094817	0.103284	0.186954	0.110619	0.062861
k__Bacteria;p__Proteobacteria;c__Betaproteobacteria;o__Rhodocyclales;f__Rhodocyclaceae;g__Sterolibacterium;s__	0	0	0	0	0.099199	0.173384	0.240867	0	0	0	0.101605	0.129056	0.062009
k__Bacteria;p__Proteobacteria;c__Deltaproteobacteria;o__Myxococcales;f__mle1-27;g__;s__	0.0642719	0	0.138269	0	0.072491	0.160697	0.075829	0.057713	0.066723	0	0	0.092183	0.060681
k__Bacteria;p__Firmicutes;c__Clostridia;o__Clostridiales;f__Lachnospiraceae;g__Blautia;s__	0.0892666	0	0	0.064933	0.144983	0.160697	0.169499	0.074688	0	0	0	0	0.058672
k__Bacteria;p__Cyanobacteria;c__Melainabacteria;o__Obscuribacterales;f__;g__;s__	0.0928372	0.10351	0.109987	0.116339	0	0	0	0	0.045652	0.082628	0.109734	0.04056	0.058437

k__Bacteria;p__Proteobacteria;c__Gammaproteobacteria;o__Xanthomonadales;f__CCM19a;g__CCM19a;s__	0.0749839	0.05646	0	0	0.072491	0	0	0.142586	0.112375	0.086759	0.056899	0.09587	0.058202
k__Bacteria;p__Bacteroidetes;c__Cytophagia;o__Cytophagales;f__Cytophagaceae;g__Flexibacter;s__	0.2213811	0.128603	0.094274	0.081167	0	0	0	0.040739	0.119399	0	0	0	0.05713
k__Bacteria;p__Verrucomicrobia;c__Verrucomicrobiae;o__Verrucomicrobiales;f__Verrucomicrobiaceae;g__uncultured;s__	0.0892666	0	0.078562	0.075756	0.053415	0.088806	0	0	0.084282	0.078496	0.060963	0.073746	0.056941
k__Bacteria;p__Proteobacteria;c__Betaproteobacteria;o__Burkholderiales;f__Burkholderiaceae;g__Cupriavidus;s__	0.2749411	0	0	0	0	0	0	0.098452	0	0	0.207275	0.099558	0.056685
k__Bacteria;p__Acidobacteria;c__Holophagae;o__Subgroup 7;f__;g__;s__	0.0856959	0.050187	0.072277	0.056817	0	0	0	0.067898	0.087793	0.165255	0.093477	0	0.056617
k__Bacteria;p__Planctomycetes;c__BD7-11;o__;f__;g__;s__	0.1428265	0.012547	0.043995	0	0	0	0	0.023764	0.059699	0.144598	0.121926	0.129056	0.056534
k__Bacteria;p__Proteobacteria;c__Betaproteobacteria;o__Betaproteobacteria Incertae Sedis;f__Unknown Family;g__Chitinivorax;s__	0	0.062733	0	0	0	0	0.115973	0.078083	0.129934	0.144598	0	0.132743	0.055339
k__Bacteria;p__Verrucomicrobia;c__Verrucomicrobiae;o__Verrucomicrobiales;f__Verrucomicrobiaceae;__;__	0.0678426	0.03764	0.072277	0.148805	0	0.177612	0	0.135796	0	0	0	0	0.053331
k__Bacteria;p__Proteobacteria;c__Alphaproteobacteria;o__Rhizobiales;f__Nordellaceae;g__JG35-K1-AG5;s__	0.1142612	0	0.103702	0	0.110645	0.135324	0	0	0.098328	0	0	0.066372	0.052386
k__Bacteria;p__SM2F11;c__;o__;f__;g__;s__	0.0999786	0.228977	0.056565	0	0	0	0	0	0	0.037182	0.142248	0.047935	0.051074
k__Bacteria;p__Spirochaetae;c__Spirochaetes;o__Spirochaetales;f__Leptospiraceae;g__uncultured;s__	0.0607013	0.06587	0.059707	0	0	0	0	0.067898	0.084282	0.14873	0.117862	0	0.050421
k__Bacteria;p__Verrucomicrobia;c__Verrucomicrobiae;o__Verrucomicrobiales;f__Verrucomicrobiaceae;g__Luteolibacter;s__	0	0.100373	0	0.216444	0	0.173384	0	0	0	0	0	0.103245	0.049454
k__Bacteria;p__Actinobacteria;c__Acidimicrobiia;o__Acidimicrobiales;f__Microthricaceae;__;__	0	0.062733	0.100559	0.089283	0	0.080348	0.156118	0.033949	0	0	0	0.062684	0.048806
k__Bacteria;p__Proteobacteria;c__Deltaproteobacteria;o__Myxococcales;f__Blfdi19;g__;s__	0.2178105	0.040777	0	0.021644	0.053415	0.054975	0.08921	0.040739	0	0	0.040642	0.022124	0.048445
k__Bacteria;p__Firmicutes;c__Bacilli;o__Bacillales;f__Bacillaceae;g__Bacillus;s__	0.0607013	0.05646	0	0.062228	0	0	0.223025	0	0	0	0.073156	0.088496	0.047005
k__Bacteria;p__Bacteroidetes;c__Flavobacteriia;o__Flavobacteriales;f__Flavobacteriaceae;g__Ornithobacterium;s__	0.0678426	0.050187	0	0.059522	0	0.088806	0.17396	0.047529	0.042141	0.033051	0	0	0.04692
k__Bacteria;p__Candidate division WS6;c__;o__;f__;g__;s__	0.0464186	0.07528	0.047137	0.054111	0.17169	0.080348	0	0.078083	0	0	0	0	0.046089
k__Bacteria;p__Proteobacteria;c__Betaproteobacteria;o__Burkholderiales;f__Comamonadaceae;g__Leptothrix;s__	0	0.106647	0.131984	0	0.209844	0	0	0	0.10184	0	0	0	0.04586
k__Bacteria;p__Proteobacteria;c__Betaproteobacteria;o__Burkholderiales;__;__;__	0.1678212	0.090963	0.028282	0	0	0	0	0	0	0	0.138183	0.117994	0.04527
k__Bacteria;p__Proteobacteria;c__Alphaproteobacteria;o__Rickettsiales;f__Holosporaceae;g__uncultured;s__	0.2320931	0	0	0.162333	0	0	0	0	0	0	0.146312	0	0.045062
k__Bacteria;p__Chloroflexi;c__Anaerolineae;o__Anaerolineales;f__Anaerolineaceae;g__H39;s__	0	0.08469	0.106844	0	0.091568	0	0	0	0.147493	0	0.093477	0.014749	0.044902
k__Bacteria;p__Proteobacteria;c__Gammaproteobacteria;o__Thiotrichales;f__Thiotrichaceae;g__Thiothrix;s__	0	0.106647	0.07542	0.108222	0	0	0.08921	0	0	0	0.073156	0.081121	0.044481
k__Bacteria;p__Acidobacteria;c__Acidobacteria;o__Subgroup 3;f__SJA-149;g__;s__	0	0	0	0.086578	0.072491	0	0	0.15956	0.073746	0.111547	0	0	0.041994
k__Bacteria;p__Proteobacteria;c__Gammaproteobacteria;o__Methylococcales;__;__;__	0	0	0	0	0	0	0	0	0	0.177649	0.170697	0.15118	0.041627
k__Bacteria;p__Proteobacteria;c__Alphaproteobacteria;o__Rhizobiales;f__Hyphomicrobiaceae;__;__	0	0.24466	0.028282	0	0	0	0	0	0	0	0	0.221239	0.041182
k__Bacteria;p__Proteobacteria;c__Alphaproteobacteria;o__Rhodobacterales;f__Rhodobacteraceae;__;__	0	0.26348	0.207404	0	0	0	0	0	0	0	0	0	0.03924
k__Bacteria;p__Proteobacteria;c__Gammaproteobacteria;o__Aeromonadales;f__Aeromonadaceae;g__Aeromonas;s__	0	0.172517	0	0	0.106829	0	0	0	0	0.181781	0	0	0.038427
k__Bacteria;p__Proteobacteria;c__Alphaproteobacteria;o__Rickettsiales;f__LWSR-14;g__;s__	0.1392559	0.163107	0.047137	0	0	0.046518	0	0	0	0	0	0.033186	0.035767
k__Bacteria;p__Firmicutes;c__Erysipelotrichia;o__Erysipelotrichales;f__Erysipelotrichaceae;g__Turicibacter;s__	0.0571306	0.072143	0.097417	0.078461	0	0.063433	0	0	0	0	0	0.05531	0.035325
k__Bacteria;p__Proteobacteria;c__Alphaproteobacteria;o__Rhodobacterales;f__Rhodobacteraceae;g__Rubellimicrobium;s__	0	0.156833	0.175979	0.083872	0	0	0	0	0	0	0	0	0.034724
k__Bacteria;p__Proteobacteria;c__Gammaproteobacteria;o__NKB5;f__;g__;s__	0	0	0	0	0	0	0.17396	0.088267	0	0	0	0.147493	0.034143
k__Bacteria;p__Chloroflexi;c__Ardenticatenia;o__uncultured;f__;g__;s__	0	0.059597	0.087989	0.083872	0	0	0.049066	0	0	0	0.020321	0.106932	0.033981
k__Bacteria;p__Proteobacteria;c__SPOTSOCT00m83;o__;f__;g__;s__	0	0	0	0	0	0	0.165039	0.081477	0.087793	0	0	0.066372	0.03339
k__Bacteria;p__Proteobacteria;c__Deltaproteobacteria;o__Bdellovibrionales;f__Bacteriovoracaceae;g__Peredibacter;s__	0	0	0.182264	0	0	0	0	0.047529	0.063211	0.033051	0	0.070059	0.033009
k__Bacteria;p__Proteobacteria;c__Deltaproteobacteria;__;__;__;__	0.1178319	0.025093	0	0.051406	0	0	0	0	0	0	0.10567	0.088496	0.032375
k__Bacteria;p__Actinobacteria;c__Thermoleophilia;o__Solirubrobacterales;f__480-2;g__;s__	0	0.12233	0.097417	0.043289	0.045784	0	0	0	0.070235	0	0	0	0.031588
k__Bacteria;p__Chloroflexi;c__WCHB1-50;o__WCHB1-50;f__WCHB1-50;g__SM1F10;s__	0.05356	0.06587	0.097417	0.037878	0	0	0.053526	0	0	0.02892	0.032514	0	0.030807
k__Bacteria;p__Chloroflexi;c__SJA-15;o__C10_SB1A;f__C10_SB1A;g__Candidatus Amarilinum;s__	0.0892666	0	0	0	0.068676	0.016915	0	0	0	0	0	0.19174	0.03055
k__Bacteria;p__Chloroflexi;c__TK10;o__;f__;g__;s__	0	0.081553	0.078562	0.070344	0	0	0	0.040739	0.094817	0	0	0	0.030501
k__Bacteria;p__Bacteroidetes;c__Cytophagia;o__Cytophagales;f__Cytophagaceae;g__Leadbetterella;s__	0.0285653	0	0	0	0.080122	0.067662	0.147197	0	0.024582	0	0.016257	0	0.030365
k__Bacteria;p__Firmicutes;c__Clostridia;o__Clostridiales;f__Lachnospiraceae;g__Lachnoclostridium;s__	0.0928372	0	0.147697	0	0	0	0	0.115426	0	0	0	0	0.029663
k__Bacteria;p__Proteobacteria;c__Betaproteobacteria;o__Neisseriales;f__Neisseriaceae;g__Rivicola;s__	0.0821253	0	0	0	0	0	0	0.067898	0	0	0.073156	0.121681	0.028738
k__Bacteria;p__GOUTA4;c__;o__;f__;g__;s__	0.1106906	0.090963	0	0	0	0	0.053526	0	0	0	0	0.088496	0.02864
k__Bacteria;p__Proteobacteria;c__Deltaproteobacteria;o__Myxococcales;__;__;__	0	0	0	0	0.068676	0	0	0.115426	0	0.070233	0.081284	0	0.027968
k__Bacteria;p__Proteobacteria;c__Deltaproteobacteria;o__Myxococcales;f__KD3-10;g__;s__	0.1356852	0	0	0	0	0	0.057987	0.084872	0	0	0	0.05531	0.027821
k__Bacteria;p__Proteobacteria;c__Alphaproteobacteria;o__Rhizobiales;f__Nordellaceae;g__Nordella;s__	0.2285225	0	0.103702	0	0	0	0	0	0	0	0	0	0.027685
k__Bacteria;p__Bacteroidetes;c__Sphingobacteriia;o__Sphingobacteriales;f__Saprospiraceae;__;__	0	0	0.02514	0	0	0	0	0.061108	0	0.14873	0.093477	0	0.027371
k__Bacteria;p__Bacteroidetes;c__Sphingobacteriia;o__Sphingobacteriales;f__Chitinophagaceae;g__Hydrotalea;s__	0	0	0	0	0	0	0.316696	0	0	0	0	0	0.026391
k__Bacteria;p__Proteobacteria;c__Alphaproteobacteria;o__Rhizobiales;f__Hyphomicrobiaceae;g__uncultured;s__	0	0	0.103702	0.0487	0	0	0	0.067898	0.049164	0	0.040642	0	0.025842
k__Bacteria;p__Lentisphaerae;__;__;__;__;__	0.042848	0.069007	0	0	0	0.080348	0	0	0	0.061971	0.052835	0	0.025584
k__Bacteria;p__Elusimicrobia;c__Elusimicrobia;o__Lineage IIb;f__;g__;s__	0	0	0	0	0	0	0	0.061108	0	0	0.142248	0.103245	0.02555
k__Bacteria;p__Planctomycetes;c__Planctomycetacia;o__Planctomycetales;f__Planctomycetaceae;__;__	0.0642719	0	0.097417	0.018939	0	0	0	0.118821	0	0	0	0	0.024954
k__Bacteria;p__Fusobacteria;c__Fusobacteriia;o__Fusobacteriales;f__Leptotrichiaceae;g__Streptobacillus;s__	0	0.02823	0.03771	0.037878	0.053415	0.093035	0	0	0.014047	0.02892	0	0	0.024436
k__Bacteria;p__Firmicutes;c__Bacilli;o__Bacillales;f__Staphylococcaceae;g__Staphylococcus;s__	0	0	0	0	0	0	0.289933	0	0	0	0	0	0.024161
k__Bacteria;p__Bacteroidetes;c__Sphingobacteriia;o__Sphingobacteriales;f__Saprospiraceae;g__Phaeodactylibacter;s__	0.0749839	0.021957	0	0	0	0.059204	0.044605	0.030554	0.056188	0	0	0	0.023958
k__Bacteria;p__Proteobacteria;c__Deltaproteobacteria;o__Myxococcales;f__bacteriap25;g__;s__	0.0249946	0.015683	0	0.154217	0	0	0	0	0	0	0	0.092183	0.023923
k__Bacteria;p__Actinobacteria;c__Actinobacteria;o__PeM15;f__PeM15;g__rJ14;s__	0	0	0.047137	0.059522	0.061045	0	0.115973	0	0	0	0	0	0.02364
k__Bacteria;p__Fusobacteria;c__Fusobacteriia;o__Fusobacteriales;f__Leptotrichiaceae;g__uncultured;s__	0	0	0	0	0	0	0.281012	0	0	0	0	0	0.023418
k__Bacteria;p__Actinobacteria;c__Acidimicrobiia;o__Acidimicrobiales;__;__;__	0	0.078417	0	0	0	0	0	0	0.084282	0.053708	0	0.058997	0.02295
k__Bacteria;p__Proteobacteria;c__Betaproteobacteria;o__Burkholderiales;f__Oxalobacteraceae;g__Undibacterium;s__	0	0	0.273396	0	0	0	0	0	0	0	0	0	0.022783
k__Bacteria;p__Bacteroidetes;c__Flavobacteriia;o__Flavobacteriales;f__Cryomorphaceae;g__Crocinitomix;s__	0.0714133	0	0	0	0.015261	0	0.08921	0	0	0.024788	0.032514	0.036873	0.022505
k__Bacteria;p__Bacteroidetes;c__Cytophagia;o__Cytophagales;f__Cytophagaceae;g__Runella;s__	0.0678426	0.034503	0.028282	0.045994	0.053415	0	0	0	0	0.037182	0	0	0.022268
k__Bacteria;p__Verrucomicrobia;__;__;__;__;__	0	0.053323	0	0	0	0	0	0.061108	0	0.09089	0	0.044248	0.020797
k__Bacteria;p__Actinobacteria;c__Actinobacteria;o__Frankiales;f__Cryptosporangiaceae;g__Fodinicola;s__	0	0	0	0	0	0.245274	0	0	0	0	0	0	0.02044
k__Bacteria;p__Bacteroidetes;c__Flavobacteriia;o__Flavobacteriales;f__Cryomorphaceae;g__Fluviicola;s__	0	0.01882	0	0	0	0.054975	0.08475	0	0	0.024788	0.024385	0.029499	0.019768
k__Bacteria;p__Actinobacteria;c__Thermoleophilia;o__Gaiellales;f__uncultured;g__;s__	0	0.07528	0	0	0	0	0.156118	0	0	0	0	0	0.019283
k__Bacteria;p__Proteobacteria;c__Gammaproteobacteria;o__Cellvibrionales;f__Cellvibrionaceae;g__Simiduia;s__	0.0892666	0	0	0	0	0	0.138276	0	0	0	0	0	0.018962
k__Bacteria;p__Proteobacteria;c__Alphaproteobacteria;o__Rickettsiales;f__AKIW1012;g__;s__	0	0.087827	0.07542	0	0	0	0.057987	0	0	0	0	0	0.018436
k__Bacteria;p__Planctomycetes;c__Planctomycetacia;o__Planctomycetales;f__Planctomycetaceae;g__Isosphaera;s__	0.0499893	0	0	0.094694	0.076307	0	0	0	0	0	0	0	0.018416
k__Bacteria;p__Proteobacteria;c__Betaproteobacteria;o__Burkholderiales;f__Comamonadaceae;g__Sphaerotilus;s__	0.2178105	0	0	0	0	0	0	0	0	0	0	0	0.018151
k__Bacteria;p__Bacteroidetes;c__Sphingobacteriia;o__Sphingobacteriales;f__LiUU-11-161;g__;s__	0	0	0	0	0	0	0.214104	0	0	0	0	0	0.017842
k__Bacteria;p__Acidobacteria;c__Holophagae;o__Subgroup 10;f__ABS-19;g__B1-K1A-105;s__	0	0	0.031425	0	0	0	0	0	0.10184	0	0.069092	0	0.016863
k__Bacteria;p__Acidobacteria;c__Acidobacteria;o__Subgroup 3;f__Unknown Family;g__Candidatus Solibacter;s__	0	0	0	0	0	0	0.196262	0	0	0	0	0	0.016355
k__Bacteria;p__Planctomycetes;c__Phycisphaerae;o__mle1-8;f__;g__;s__	0	0	0	0	0	0	0	0	0	0	0.138183	0.051622	0.015817
k__Bacteria;p__Bacteroidetes;c__Bacteroidia;o__Bacteroidales;f__Porphyromonadaceae;g__Microbacter;s__	0	0	0	0	0.072491	0.059204	0.057987	0	0	0	0	0	0.015807
k__Bacteria;p__Proteobacteria;c__Deltaproteobacteria;o__Myxococcales;f__Cystobacteraceae;g__Anaeromyxobacter;s__	0.032136	0	0	0	0	0	0	0.067898	0.052676	0	0.036578	0	0.015774
k__Bacteria;p__Deferribacteres;c__Deferribacteres Incertae Sedis;o__Unknown Order;f__Unknown Family;g__Caldithrix;s__	0	0	0	0	0	0.029602	0	0.098452	0.059699	0	0	0	0.015646
k__Bacteria;p__Proteobacteria;c__Alphaproteobacteria;o__Rhizobiales;f__Bradyrhizobiaceae;g__uncultured;s__	0	0	0.116272	0.067639	0	0	0	0	0	0	0	0	0.015326
k__Bacteria;p__Proteobacteria;c__Betaproteobacteria;o__Methylophilales;f__Methylophilaceae;__;__	0	0	0	0	0	0	0.17842	0	0	0	0	0	0.014868
k__Bacteria;p__Proteobacteria;c__Gammaproteobacteria;o__Legionellales;f__Coxiellaceae;g__Aquicella;s__	0	0	0	0	0	0	0.075829	0.101847	0	0	0	0	0.014806
k__Bacteria;p__Acidobacteria;c__Acidobacteria;o__Subgroup 4;__;__;__	0	0	0.018855	0	0	0	0	0.030554	0	0	0.125991	0	0.014617
k__Bacteria;p__Chloroflexi;__;__;__;__;__	0	0	0	0.17045	0	0	0	0	0	0	0	0	0.014204
k__Bacteria;p__Actinobacteria;c__Coriobacteriia;o__Coriobacteriales;f__Coriobacteriaceae;g__Collinsella;s__	0	0	0	0	0	0.169155	0	0	0	0	0	0	0.014096
k__Bacteria;p__Firmicutes;c__Clostridia;o__Clostridiales;f__Ruminococcaceae;g__Ruminococcus 2;s__	0	0	0.069135	0	0	0	0.098131	0	0	0	0	0	0.013939
k__Bacteria;p__Planctomycetes;c__Planctomycetacia;o__Planctomycetales;f__Planctomycetaceae;g__Singulisphaera;s__	0	0	0.163409	0	0	0	0	0	0	0	0	0	0.013617
k__Bacteria;p__Proteobacteria;c__Gammaproteobacteria;o__Xanthomonadales;f__Competibacteraceae;g__CPB_S18;s__	0	0	0	0	0	0	0	0	0	0	0	0.162242	0.01352
k__Bacteria;p__Bacteroidetes;c__SM1A07;o__;f__;g__;s__	0	0.040777	0	0	0	0	0	0	0.017559	0.041314	0.02845	0.033186	0.01344
k__Bacteria;p__Proteobacteria;c__Gammaproteobacteria;o__Aeromonadales;f__Aeromonadaceae;g__Tolumonas;s__	0	0	0	0	0	0.160697	0	0	0	0	0	0	0.013391
k__Bacteria;p__Firmicutes;c__Clostridia;o__Clostridiales;f__Lachnospiraceae;g__Anaerostipes;s__	0	0	0	0	0	0	0.156118	0	0	0	0	0	0.01301
k__Bacteria;p__Proteobacteria;c__Betaproteobacteria;o__Burkholderiales;f__Comamonadaceae;g__Rubrivivax;s__	0	0	0	0	0	0	0	0	0.154516	0	0	0	0.012876
k__Bacteria;p__Chloroflexi;c__Caldilineae;o__Caldilineales;f__Caldilineaceae;g__Litorilinea;s__	0	0	0	0.116339	0.038153	0	0	0	0	0	0	0	0.012874
k__Bacteria;p__Bacteroidetes;c__Bacteroidia;o__Bacteroidales;f__Bacteroidaceae;g__Bacteroides;s__	0	0	0	0	0	0.14801	0	0	0	0	0	0	0.012334
k__Bacteria;p__Proteobacteria;c__Betaproteobacteria;o__Neisseriales;f__Neisseriaceae;g__uncultured;s__	0	0	0	0	0	0.046518	0.075829	0	0	0.024788	0	0	0.012261
k__Bacteria;p__Firmicutes;c__Negativicutes;o__Selenomonadales;f__Veillonellaceae;g__Megamonas;s__	0	0	0	0	0.064861	0	0.080289	0	0	0	0	0	0.012096
k__Bacteria;p__Firmicutes;c__Clostridia;o__Clostridiales;f__Lachnospiraceae;g__Lachnospiraceae ND3007 group;s__	0	0	0	0	0	0	0.138276	0	0	0	0	0	0.011523
k__Bacteria;p__Planctomycetes;c__Planctomycetacia;o__Planctomycetales;f__Planctomycetaceae;g__Candidatus Nostocoida;s__	0	0.138013	0	0	0	0	0	0	0	0	0	0	0.011501
k__Bacteria;p__Proteobacteria;c__Alphaproteobacteria;o__Rickettsiales;f__SM2D12;g__;s__	0	0	0	0	0	0	0.057987	0.074688	0	0	0	0	0.011056
k__Bacteria;p__Proteobacteria;c__Gammaproteobacteria;o__Xanthomonadales;f__Xanthomonadaceae;__;__	0	0.13174	0	0	0	0	0	0	0	0	0	0	0.010978
k__Bacteria;p__Actinobacteria;c__Thermoleophilia;o__Gaiellales;f__BVA77;g__LF_BF07;s__	0	0.043913	0	0.040583	0.045784	0	0	0	0	0	0	0	0.010857
k__Bacteria;p__Cyanobacteria;c__Chloroplast;o__;f__;g__;s__	0	0	0	0	0	0	0	0	0	0.074365	0	0.05531	0.010806
k__Bacteria;p__Proteobacteria;c__Deltaproteobacteria;o__Sh765B-TzT-29;f__;g__;s__	0.042848	0	0	0	0.030523	0	0	0	0	0	0	0.05531	0.010723
k__Bacteria;p__Chloroflexi;c__WCHB1-50;o__WCHB1-50;f__WCHB1-50;g__;s__	0	0	0	0.016233	0	0	0	0	0	0	0	0.110619	0.010571
k__Bacteria;p__Firmicutes;c__Clostridia;o__Halanaerobiales;f__Halanaerobiaceae;g__Halocella;s__	0	0	0	0	0	0	0.124894	0	0	0	0	0	0.010408
k__Bacteria;p__Cyanobacteria;c__Melainabacteria;o__Vampirovibrionales;f__;g__;s__	0	0	0	0	0	0	0	0.050923	0.02107	0	0.052835	0	0.010402
k__Bacteria;p__Proteobacteria;c__Deltaproteobacteria;o__Myxococcales;f__Sandaracinaceae;__;__	0	0	0	0	0	0	0	0	0.035117	0	0	0.088496	0.010301
k__Bacteria;p__Planctomycetes;c__Phycisphaerae;o__Phycisphaerales;f__Phycisphaeraceae;g__I-8;s__	0	0	0	0	0.038153	0	0	0	0	0	0	0.084808	0.010247
k__Bacteria;p__Proteobacteria;c__Betaproteobacteria;o__Burkholderiales;f__Comamonadaceae;g__Comamonas;s__	0	0	0	0	0	0	0.120434	0	0	0	0	0	0.010036
k__Bacteria;p__Gemmatimonadetes;c__Gemmatimonadetes;o__Gemmatimonadales;f__Gemmatimonadaceae;g__Gemmatimonas;s__	0	0	0.078562	0	0	0	0	0	0.038629	0	0	0	0.009766
k__Bacteria;p__Gemmatimonadetes;c__Gemmatimonadetes;o__Gemmatimonadales;f__Gemmatimonadaceae;g__H106;s__	0	0	0	0	0	0	0	0.044134	0.070235	0	0	0	0.009531
k__Bacteria;p__Proteobacteria;c__Deltaproteobacteria;o__Bdellovibrionales;f__Bacteriovoracaceae;g__Bacteriovorax;s__	0	0	0	0	0	0	0.111513	0	0	0	0	0	0.009293
k__Bacteria;p__Bacteroidetes;c__Flavobacteriia;o__Flavobacteriales;f__Flavobacteriaceae;g__Bergeyella;s__	0.1106906	0	0	0	0	0	0	0	0	0	0	0	0.009224
k__Bacteria;p__Firmicutes;c__Clostridia;o__Clostridiales;f__Lachnospiraceae;g__Incertae Sedis;s__	0	0	0	0	0.110645	0	0	0	0	0	0	0	0.00922
k__Bacteria;p__Chlamydiae;c__Chlamydiae;o__Chlamydiales;f__Simkaniaceae;g__Candidatus Rhabdochlamydia;s__	0.0357066	0	0	0	0	0	0	0	0	0	0.073156	0	0.009072

k__Bacteria;p__Planctomycetes;c__028H05-P-BN-P5;o__;f__;g__;s__	0	0	0	0	0	0	0	0.047529	0	0	0.056899	0	0.008702
k__Bacteria;p__Bacteroidetes;c__Cytophagia;o__Cytophagales;f__Cytophagaceae;g__Sporocytophaga;s__	0	0	0	0	0	0	0.102592	0	0	0	0	0	0.008549
k__Bacteria;p__Cyanobacteria;c__ML635J-21;o__;f__;g__;s__	0.032136	0.025093	0	0	0	0	0	0	0	0.016526	0.02845	0	0.008517
k__Bacteria;p__Planctomycetes;__;__;__;__;__	0.042848	0	0	0	0	0	0	0	0	0	0	0.05531	0.00818
k__Bacteria;p__Proteobacteria;c__Alphaproteobacteria;o__Rhizobiales;f__Bradyrhizobiaceae;g__Afipia;s__	0	0	0	0.0974	0	0	0	0	0	0	0	0	0.008117
k__Bacteria;p__Proteobacteria;c__Gammaproteobacteria;o__Pseudomonadales;f__Moraxellaceae;g__Moraxella;s__	0	0	0	0.0487	0	0.046518	0	0	0	0	0	0	0.007935
k__Bacteria;p__Spirochaetae;c__Spirochaetes;o__Spirochaetales;f__Spirochaetaceae;g__Spirochaeta 2;s__	0	0	0	0	0	0	0.093671	0	0	0	0	0	0.007806
k__Bacteria;p__Chlamydiae;c__Chlamydiae;o__Chlamydiales;__;__;__	0.0928372	0	0	0	0	0	0	0	0	0	0	0	0.007736
k__Bacteria;p__Acidobacteria;c__Acidobacteria;o__Subgroup 17;f__CCU21;g__;s__	0	0.090963	0	0	0	0	0	0	0	0	0	0	0.00758
k__Bacteria;p__Deinococcus-Thermus;c__Deinococci;o__Deinococcales;f__Deinococcaceae;g__Deinococcus;s__	0	0	0	0	0	0.050746	0.040145	0	0	0	0	0	0.007574
k__Bacteria;p__Bacteroidetes;c__Cytophagia;o__Order II;f__Rhodothermaceae;g__;s__	0	0	0.047137	0	0	0	0	0	0.014047	0	0	0.029499	0.007557
k__Bacteria;p__Chlorobi;c__Ignavibacteria;o__Ignavibacteriales;f__BSV26;g__;s__	0.0464186	0	0	0	0	0	0	0	0	0	0.040642	0	0.007255
k__Bacteria;p__Proteobacteria;c__Alphaproteobacteria;o__Rhizobiales;f__Rhizobiaceae;g__Shinella;s__	0.0856959	0	0	0	0	0	0	0	0	0	0	0	0.007141
k__Bacteria;p__Proteobacteria;c__Alphaproteobacteria;o__Rickettsiales;f__Rickettsiales Incertae Sedis;__;__	0	0	0	0	0	0	0	0	0	0	0.085349	0	0.007112
k__Bacteria;p__Proteobacteria;c__Deltaproteobacteria;o__Desulfobacterales;f__Desulfobulbaceae;__;__	0	0	0	0	0	0	0.08475	0	0	0	0	0	0.007062
k__Bacteria;p__Proteobacteria;c__Betaproteobacteria;o__Burkholderiales;f__Comamonadaceae;g__Caenimonas;s__	0	0	0	0.083872	0	0	0	0	0	0	0	0	0.006989
k__Bacteria;p__Proteobacteria;c__Alphaproteobacteria;o__Rhodospirillales;f__Rhodospirillaceae;g__Inquilinus;s__	0	0.034503	0.021997	0.027056	0	0	0	0	0	0	0	0	0.006963
k__Bacteria;p__Chlamydiae;c__Chlamydiae;o__Chlamydiales;f__Parachlamydiaceae;g__Neochlamydia;s__	0.0821253	0	0	0	0	0	0	0	0	0	0	0	0.006844
k__Bacteria;p__Proteobacteria;c__Alphaproteobacteria;o__Rhizobiales;f__Hyphomicrobiaceae;g__Pedomicrobium;s__	0	0.081553	0	0	0	0	0	0	0	0	0	0	0.006796
k__Bacteria;p__Proteobacteria;c__Alphaproteobacteria;o__Caulobacterales;f__Hyphomonadaceae;g__Hirschia;s__	0	0	0	0	0	0	0.040145	0	0	0.041314	0	0	0.006788
k__Bacteria;p__Proteobacteria;c__Betaproteobacteria;o__Rhodocyclales;f__Rhodocyclaceae;g__Azovibrio;s__	0	0	0.028282	0	0	0	0	0	0	0	0.052835	0	0.00676
k__Bacteria;p__Chloroflexi;c__KD4-96;o__;f__;g__;s__	0	0	0	0	0	0	0.026763	0.054318	0	0	0	0	0.006757
k__Bacteria;p__Proteobacteria;c__Betaproteobacteria;o__Burkholderiales;f__Alcaligenaceae;__;__	0	0	0	0	0	0	0.080289	0	0	0	0	0	0.006691
k__Bacteria;p__Actinobacteria;c__Actinobacteria;o__Actinomycetales;f__Actinomycetaceae;g__uncultured;s__	0	0	0	0.078461	0	0	0	0	0	0	0	0	0.006538
k__Bacteria;p__Acidobacteria;c__Acidobacteria;o__Subgroup 4;f__RB41;g__;s__	0	0	0	0	0	0	0.075829	0	0	0	0	0	0.006319
k__Bacteria;p__Fibrobacteres;c__Fibrobacteria;o__Fibrobacterales;f__Fibrobacteraceae;g__uncultured;s__	0	0	0	0	0	0.025373	0.049066	0	0	0	0	0	0.006203
k__Bacteria;p__Bacteroidetes;c__Bacteroidia;o__Bacteroidales;f__Porphyromonadaceae;g__Macellibacteroides;s__	0	0	0	0	0	0	0	0	0	0	0.073156	0	0.006096
k__Bacteria;p__Proteobacteria;c__Alphaproteobacteria;o__Rhizobiales;f__Meganemaceae;g__;s__	0	0	0	0.07305	0	0	0	0	0	0	0	0	0.006087
k__Bacteria;p__Firmicutes;c__Negativicutes;o__Selenomonadales;f__Veillonellaceae;g__Veillonella;s__	0	0	0	0	0	0	0.071368	0	0	0	0	0	0.005947
k__Bacteria;p__Chloroflexi;c__Ardenticatenia;o__419;f__2-1;g__Candidatus Promineofilum;s__	0	0	0.069135	0	0	0	0	0	0	0	0	0	0.005761
k__Bacteria;p__Proteobacteria;c__Gammaproteobacteria;o__Enterobacteriales;f__Enterobacteriaceae;__;__	0	0	0	0	0.068676	0	0	0	0	0	0	0	0.005723
k__Bacteria;p__Proteobacteria;c__Betaproteobacteria;o__Burkholderiales;f__Comamonadaceae;g__Rhizobacter;s__	0	0	0	0	0	0	0	0	0	0	0	0.066372	0.005531
k__Bacteria;p__Proteobacteria;c__Alphaproteobacteria;__;__;__;__	0	0.034503	0.028282	0	0	0	0	0	0	0	0	0	0.005232
k__Bacteria;p__Proteobacteria;c__Alphaproteobacteria;o__Rickettsiales;f__Rickettsiales Incertae Sedis;g__Candidatus Odyssella;s__	0	0	0	0	0	0	0.062447	0	0	0	0	0	0.005204
k__Bacteria;p__Proteobacteria;c__Gammaproteobacteria;o__Thiotrichales;f__Thiotrichaceae;g__Beggiatoa;s__	0	0	0	0	0	0	0.062447	0	0	0	0	0	0.005204
k__Bacteria;p__Firmicutes;c__Negativicutes;o__Selenomonadales;f__Veillonellaceae;g__Dialister;s__	0	0	0.03771	0	0	0	0	0.023764	0	0	0	0	0.005123
k__Bacteria;p__Acidobacteria;c__Holophagae;o__Holophagales;f__Holophagaceae;g__Geothrix;s__	0	0.031367	0.028282	0	0	0	0	0	0	0	0	0	0.004971
k__Bacteria;p__Proteobacteria;c__Gammaproteobacteria;o__Chromatiales;f__Ectothiorhodospiraceae;g__Thioalkalispira;s__	0	0	0	0	0	0	0	0	0	0	0.056899	0	0.004742
k__Bacteria;p__Proteobacteria;c__Deltaproteobacteria;o__Myxococcales;f__Sandaracinaceae;g__;s__	0	0	0.015712	0	0	0	0	0	0	0	0	0.04056	0.004689
k__Bacteria;p__Chlamydiae;c__Chlamydiae;o__Chlamydiales;f__cvE6;g__;s__	0	0	0	0	0	0	0	0	0.056188	0	0	0	0.004682
k__Bacteria;p__Bacteroidetes;c__Bacteroidia;o__Bacteroidales;f__Rikenellaceae;g__vadinBC27 wastewater-sludge group;s__	0	0	0	0	0	0	0.053526	0	0	0	0	0	0.004461
k__Bacteria;p__Verrucomicrobia;c__Opitutae;o__Opitutae vadinHA64;f__;g__;s__	0	0	0	0	0	0	0.053526	0	0	0	0	0	0.004461
k__Bacteria;p__Proteobacteria;c__Deltaproteobacteria;o__Myxococcales;f__Polyangiaceae;__;__	0	0.053323	0	0	0	0	0	0	0	0	0	0	0.004444
k__Bacteria;p__Proteobacteria;c__Gammaproteobacteria;o__Legionellales;f__Legionellaceae;g__Legionella;s__	0	0	0	0	0	0	0	0	0	0	0.052835	0	0.004403
k__Bacteria;p__Planctomycetes;c__Phycisphaerae;o__Pla1 lineage;f__;g__;s__	0	0	0	0	0	0	0	0	0.052676	0	0	0	0.00439
k__Bacteria;p__Actinobacteria;c__Actinobacteria;o__Frankiales;f__Geodermatophilaceae;g__c22;s__	0	0	0	0.051406	0	0	0	0	0	0	0	0	0.004284
k__Bacteria;p__Proteobacteria;c__Betaproteobacteria;o__SC-I-84;f__A21b;g__;s__	0	0	0	0	0	0	0	0.050923	0	0	0	0	0.004244
k__Bacteria;p__Omnitrophica;c__NPL-UPA2;o__;f__;g__;s__	0	0	0	0	0	0	0	0	0	0.049577	0	0	0.004131
k__Bacteria;p__Firmicutes;c__Clostridia;o__Clostridiales;f__Ruminococcaceae;g__Ruminococcaceae UCG-014;s__	0	0	0	0	0	0	0.049066	0	0	0	0	0	0.004089
k__Bacteria;p__Microgenomates;c__;o__;f__;g__;s__	0	0	0	0.045994	0	0	0	0	0	0	0	0	0.003833
k__Bacteria;p__Proteobacteria;c__Gammaproteobacteria;o__Methylococcales;f__Methylococcaceae;g__Methylocaldum;s__	0	0	0	0.045994	0	0	0	0	0	0	0	0	0.003833
k__Bacteria;p__Nitrospirae;c__Nitrospira;o__Nitrospirales;f__4-29;g__;s__	0	0	0	0	0.045784	0	0	0	0	0	0	0	0.003815
k__Bacteria;p__Actinobacteria;c__Actinobacteria;o__Propionibacteriales;f__Propionibacteriaceae;g__Propionibacterium;s__	0	0	0	0	0	0	0.044605	0	0	0	0	0	0.003717
k__Bacteria;p__Bacteroidetes;c__Bacteroidia;o__Bacteroidales;f__Rikenellaceae;g__Alistipes;s__	0	0	0	0	0	0	0.044605	0	0	0	0	0	0.003717
k__Bacteria;p__Proteobacteria;c__Betaproteobacteria;o__Burkholderiales;f__Comamonadaceae;g__Hydrogenophaga;s__	0	0	0	0.043289	0	0	0	0	0	0	0	0	0.003607
k__Bacteria;p__Elusimicrobia;c__Elusimicrobia;o__Lineage IIa;f__;g__;s__	0.042848	0	0	0	0	0	0	0	0	0	0	0	0.003571
k__Bacteria;p__Proteobacteria;c__Betaproteobacteria;o__Hot Creek 32;f__;g__;s__	0.0071413	0	0.003142	0.010822	0.003815	0	0	0	0	0.008263	0.008128	0	0.003443
k__Bacteria;p__Planctomycetes;c__Planctomycetacia;o__Planctomycetales;f__Planctomycetaceae;g__Zavarzinella;s__	0	0	0	0	0	0	0.040145	0	0	0	0	0	0.003345
k__Bacteria;p__Acidobacteria;c__Subgroup 26;o__;f__;g__;s__	0	0	0.03771	0	0	0	0	0	0	0	0	0	0.003142
k__Bacteria;p__Planctomycetes;c__Phycisphaerae;o__WD2101 soil group;f__;g__;s__	0	0	0	0	0	0	0.035684	0	0	0	0	0	0.002974
k__Bacteria;p__Proteobacteria;c__Alphaproteobacteria;o__Rickettsiales;f__Rickettsiales Incertae Sedis;g__Candidatus Captivus;s__	0	0.034503	0	0	0	0	0	0	0	0	0	0	0.002875
k__Bacteria;p__Proteobacteria;c__Alphaproteobacteria;o__Caulobacterales;f__Caulobacteraceae;g__Phenylobacterium;s__	0	0	0	0	0.034338	0	0	0	0	0	0	0	0.002862
k__Bacteria;p__Proteobacteria;c__Alphaproteobacteria;o__Rhodospirillales;__;__;__	0	0	0	0	0	0	0	0	0.031606	0	0	0	0.002634
k__Bacteria;p__Firmicutes;c__Clostridia;o__Clostridiales;f__Ruminococcaceae;g__[Eubacterium] coprostanoligenes group;s__	0.0285653	0	0	0	0	0	0	0	0	0	0	0	0.00238
k__Bacteria;p__Planctomycetes;c__Planctomycetacia;o__Planctomycetales;f__Planctomycetaceae;g__Blastopirellula;s__	0.0285653	0	0	0	0	0	0	0	0	0	0	0	0.00238
k__Bacteria;p__Bacteroidetes;c__Bacteroidia;o__Bacteroidales;f__Prevotellaceae;g__uncultured;s__	0	0	0	0	0	0	0	0	0	0	0.02845	0	0.002371
k__Bacteria;p__Firmicutes;c__Clostridia;o__Clostridiales;f__Ruminococcaceae;g__Ruminococcaceae UCG-002;s__	0	0.02823	0	0	0	0	0	0	0	0	0	0	0.002352
k__Bacteria;p__Fibrobacteres;c__Fibrobacteria;o__Fibrobacterales;f__Fibrobacteraceae;g__;s__	0	0	0	0	0	0	0.022303	0	0	0	0	0	0.001859
k__Bacteria;p__Chloroflexi;c__SJA-15;o__1-20;f__1-20;g__Candidatus Sarcinathrix;s__	0	0	0.021997	0	0	0	0	0	0	0	0	0	0.001833
k__Bacteria;p__Proteobacteria;c__Epsilonproteobacteria;o__Campylobacterales;f__Campylobacteraceae;g__Sulfurospirillum;s__	0	0	0	0	0	0	0	0	0	0	0.020321	0	0.001693
k__Bacteria;p__Chlamydiae;c__Chlamydiae;o__Chlamydiales;f__Chlamydiales Incertae Sedis;g__Criblamydia;s__	0	0.01882	0	0	0	0	0	0	0	0	0	0	0.001568
k__Bacteria;p__Proteobacteria;c__Alphaproteobacteria;o__Rickettsiales;__;__;__	0	0	0	0	0	0	0	0	0	0	0.016257	0	0.001355
k__Bacteria;p__Proteobacteria;c__Alphaproteobacteria;o__Rickettsiales;f__uncultured;g__;s__	0	0	0	0.016233	0	0	0	0	0	0	0	0	0.001353
k__Bacteria;p__Verrucomicrobia;c__Spartobacteria;o__Chthoniobacterales;f__Xiphinematobacteraceae;g__Candidatus Xiphinematoba	0	0	0.01257	0	0	0	0	0	0	0	0	0	0.001047
k__Bacteria;p__Firmicutes;c__Clostridia;o__Clostridiales;f__Ruminococcaceae;g__Ruminococcaceae UCG-013;s__	0.010712	0	0	0	0	0	0	0	0	0	0	0	0.000893
k__Bacteria;p__Proteobacteria;c__Alphaproteobacteria;o__Rickettsiales;f__LR A2-29;g__;s__	0	0.00941	0	0	0	0	0	0	0	0	0	0	0.000784
k__Bacteria;p__Bacteroidetes;c__Sphingobacteriia;o__Sphingobacteriales;f__WCHB1-69;g__;s__	0	0	0	0	0	0	0	0	0	0	0.008128	0	0.000677

Table S2 Coefficients, *t*-test results, and VIF values in the final multiple regression model.

Independent variable	Unstd. coefficient		Std. coefficient beta	p	VIF
	B	Std. error			
Constant	-2.31×10^4	7.81×10^4		0.771	
Temperature (°C)	-1.46×10^4	3.68×10^3	−0.71	< 0.001	2.20
SRT (d)	1.06×10^4	3.05×10^3	0.57	0.003	1.84
SS in return sludge (mg L^{-1})	3.35×10	9.87	0.52	0.004	1.640
SS (mg L^{-1})	4.46×10^3	1.34×10^3	0.43	0.004	1.124

$R = 0.867$, $R^2 = 0.752$, R^2 (adj.) = 0.693.

Note: Dependent variable: *Kouleothrix* gene copy number, VIF: Variance inflation factor.

Table S3 Analysis of variance for the final multiple regression model.

	SS	*df*	MS	*F* value	p
Regression	7.97×10^{10}	4	1.99×10^{10}	12.9	< 0.001
Residual error	2.63×10^{10}	17	1.55×10^9		
Total	1.06×10^{11}	21			

Note: SS: Sum of squares, *df*: Degree of freedom, MS: Mean square.
